# Point defect formation at finite temperatures with machine learning force fields†

**DOI:** 10.1039/d4sc08582e

**Published:** 2025-04-08

**Authors:** Irea Mosquera-Lois, Johan Klarbring, Aron Walsh

**Affiliations:** a Thomas Young Centre & Department of Materials, Imperial College London London SW7 2AZ UK a.walsh@imperial.ac.uk; b Department of Physics, Chemistry and Biology (IFM), Linköping University SE-581 83 Linköping Sweden

## Abstract

Point defects dictate the properties of many functional materials. The standard approach to modelling the thermodynamics of defects relies on a static description, where the change in Gibbs free energy is approximated by the internal energy. This approach has a low computational cost, but ignores contributions from atomic vibrations and structural configurations that can be accessed at finite temperatures. We train a machine learning force field (MLFF) to explore dynamic defect behaviour using Te^+1^_i_ and *V*^+2^_Te_ in CdTe as exemplars. We consider the different entropic contributions (*e.g.*, electronic, spin, vibrational, orientational, and configurational) and compare methods to compute the defect free energies, ranging from a harmonic treatment to a fully anharmonic approach based on thermodynamic integration. We find that metastable configurations are populated at room temperature and thermal effects increase the predicted concentration of Te^+1^_*i*_ by two orders of magnitude — and can thus significantly affect the predicted properties. Overall, our study underscores the importance of finite-temperature effects and the potential of MLFFs to model defect dynamics at both synthesis and device operating temperatures.

## Abstract 2

Predicting accurate defect concentrations by accounting for defect entropies.

## Graphical abstract

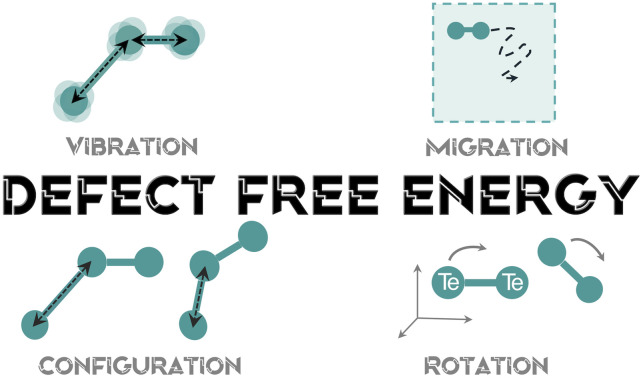

## 1. Introduction

Point defects make or break material functionality.^1^ They limit photovoltaic efficiency by acting as non-radiative recombination centres, control ionic conductivity in batteries, provide active sites for catalytic reactions, and platforms for quantum information technologies. Despite their profound effect on the macroscopic properties of crystals, they are present in dilute concentrations and thus render experimental characterisation challenging. As a result, a combination of experiment and theory is needed to understand defect behaviour.

The key factor when modelling defects is their concentration, which is determined by the free energy of defect formation, *g*_f_, at the synthesis or annealing temperature. Calculating *g*_f_ is however computationally challenging, and is thus typically approximated by the formation internal energy, *u*_f_(0 K), *i.e. g*_f_(*T*_synthesis_) ≈ *u*_f_(0 K).^2,3^ Inherent in this approximation is the assumption of a static framework, where most studies only consider the defect ground state structure at 0 K, and thus neglect metastable configurations that may be populated at the device operating temperature. Since the properties of a defect strongly depend on its geometry,^4–9^ the predicted behaviour can be significantly affected when ignoring thermally accessible metastable configurations.^10–16^

With the development of better computational resources, more accurate studies that go beyond this static 0 K approximation are becoming possible using *ab initio* methods. In the last decades, many investigations have modelled entropic contributions for defects in elementary solids.^17–30^ Thermal effects are harder to model in multinary semiconductors due to the higher number of possible intrinsic charged defects and required level of theory,^31–61^ but have been included for specific defects.^62–80^ However, most of these studies adopt several approximations: (i) they only account for vibrational entropies, thereby neglecting other degrees of freedom (*e.g.*, electronic, spin, orientational and configurational), and (ii) they adopt the (quasi)harmonic approximation to model vibrational effects (*e.g.*, assuming a quadratic potential energy surface for the interatomic bonds). The limitations of these approximations are system-dependent and not well investigated, and demonstrate the lack of a reliable and affordable approach to model thermal effects for defects.

In this study, we target these limitations by considering all relevant entropic contributions and systematically comparing the different methods to calculate the defect formation free energy, ranging from a harmonic to a fully anharmonic approach based on thermodynamic integration (TI). To reduce the cost of these simulations, we use machine learning force fields as a surrogate model, which successfully map the defect energy surfaces (Section 2.1). We choose Te^+1^_*i*_ and *V*^+2^_Te_ in CdTe as exemplar systems since they display potential energy surfaces of different complexity (*e.g.*, presence *versus* lack of low-energy metastable configurations^12^) (Section 2.2). By modelling the impact of thermal effects on their predicted defect concentrations, we find that these dominate when the defect undergoes symmetry-breaking structural reconstructions and has low-energy metastable configurations, thereby demonstrating the limitations of the idealised 0 K description (Section 2.3).

## 2. Results and discussion

### 2.1 Machine learning force fields for defects

Calculating the defect formation free energy, *g*_f_, requires modelling the defect formation reaction by computing the free energy difference between products and reactants at the synthesis temperature.^2,3^ For example, the formation of the positively charged tellurium interstitial, Te^+1^_*i*_, can be described by the defect reaction1
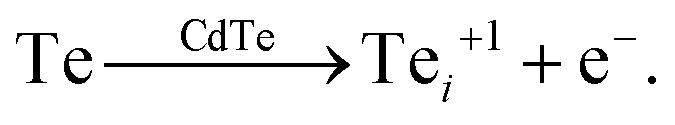


The corresponding formation free energy is defined from the sum of products (charged defect with an electron in the conduction band) minus the sum of reactants (pristine CdTe host and reservoir of Te). In the standard first-principles supercell formalism, this formation energy is given by2
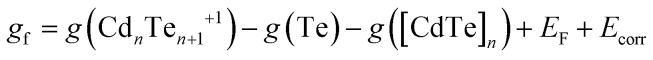
where Te represents the phase that acts as the external source of atoms during synthesis, *E*_F_ denotes the Fermi level and *E*_corr_ is the correction energy for charged defects. From these terms, the defect free energy, g(Cd_*n*_Te^+1^_*n*+1_), is the most challenging to compute due to the low symmetry and large supercells required to model defects (*e.g.*, many force calculations in the (quasi)harmonic method). This is exacerbated when going beyond the harmonic approximation since computing the anharmonic free energy with TI requires many and long molecular dynamics runs.^81,82^

**Fig. 1:**
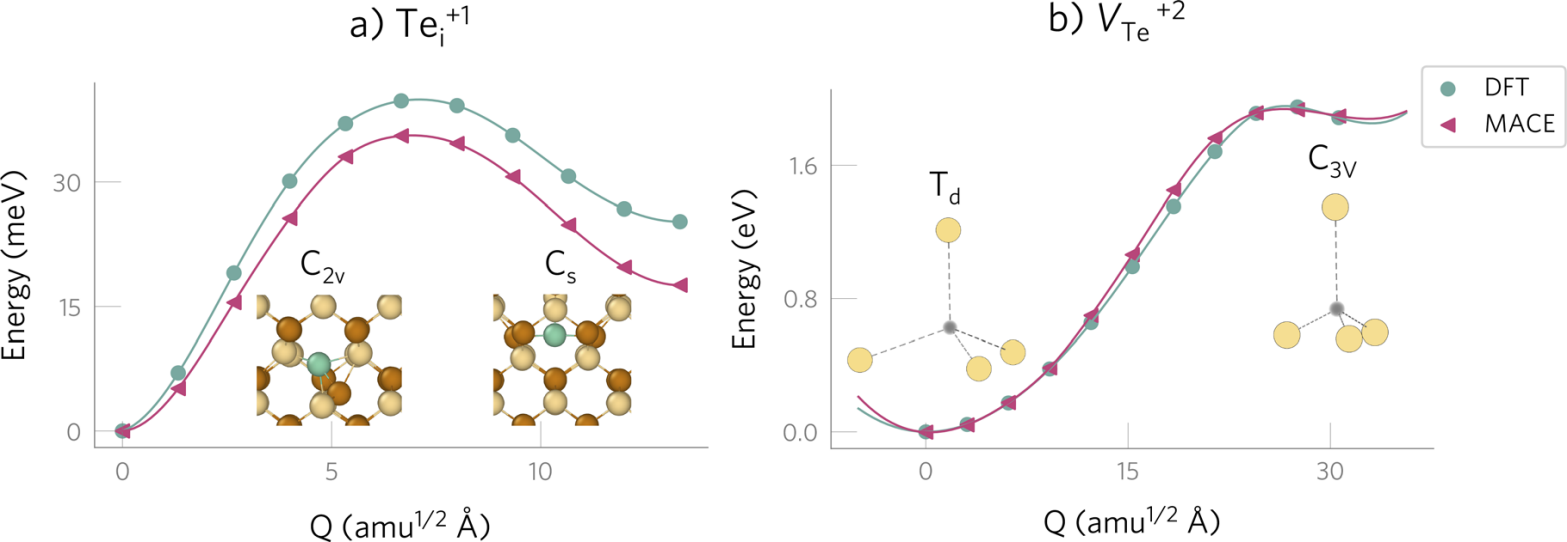
Potential energy surfaces illustrating the defect configurations identified with defect structure searching,^85^ calculated with DFT (green circles) and MACE (pink triangles). The variable *Q* represents a mass-weighted displacement coordinate that tracks the structural change along the pathway between the defect configurations, as described in the Methods. (a) Te^+1^_*i*_ is a bistable defect since the metastable configuration (split Te–Cd with *C*_s_ symmetry) is only 18 meV higher than the ground state (split Te–Te with *C*_2v_ symmetry). Note that the differences between DFT and MACE are in meV per supercell, with the error in Δ*E*(*C*_2v_ − *C*_s_) only accounting to 0.1 meV per atom. (b) *V*^+2^_Te_, where the metastable configuration (*C*_3v_) is significantly higher in energy (1.8 eV above the ground state structure of T_d_ symmetry). Te in brown, Cd in yellow, Te^+1^_*i*_ in green and *V*^+2^_Te_ in shaded grey.

To reduce the cost of free energy calculations, we employ machine learning force fields and train a separate MACE model^83^ for each system involved in the defect formation reaction, focussing on a single charge state of each defect. We target temperatures ranging from 100 K to the typical CdTe anneal temperature of 840 K.^84^ All models show good accuracies with low mean and root mean square errors on the test set (see Table 1 and further discussion in Methods and ESI†). The accuracy of the defect models is further confirmed by mapping the one-dimensional path between the stable defect structures, which shows good agreement despite the small energy difference between the distinct configurations of Te^+1^_*i*_ (Fig. 1 and ESI Fig. S9†).

**Table 1:** Mean absolute errors and root mean square errors (shown in parentheses) of the test sets for energies, forces and stresses. The relatively high errors observed for Te are caused by including its liquid phase (*T*_melt_ ≈ 704 K). Distributions of the absolute errors and the learning curve for the Te^+1^_*i*_ model are shown in the ESI

System	Energy (meV per atom)	Force (meV Å^−1^)	Stress (meV Å^−3^)
CdTe	0.3 (0.4)	13 (17)	0.2 (0.3)
Te^+1^_*i*_	0.5 (0.7)	21 (30)	0.2 (0.3)
*V* ^+2^_Te_	0.4 (0.6)	18 (24)	0.2 (0.3)
Te	1.6 (2.3)	73 (102)	0.9 (1.3)

### 2.2 Defect dynamics at room temperature

We first investigate the limitations of the static framework by comparing the behaviour of the defects at 0 K and around the typical operating temperature for a solar cell (*T* = 300 K). The potential energy surface calculated at 0 K shows that Te^+1^_*i*_ is a bistable defect with two accessible structures: a split configuration with either one or two Te–Te bonds,^12^ which have *C*_2v_ and *C*_s_ site symmetries, respectively, and an energy difference of Δ*E*(*C*_s_ − *C*_2v_) = 18 meV (Fig. 1). In contrast, *V*^+2^_Te_ only has one accessible structure at the device operating conditions since the metastable *C*_3V_ configuration is 1.8 eV above the *T*_d_ ground state (≫ *k*_B_*T* = 25 meV at 300 K).

**Fig. 2:**
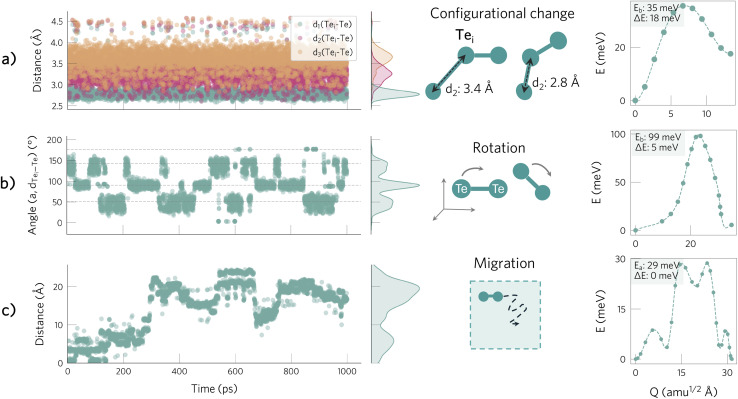
Active degrees of freedom of Te^+1^_*i*_ at 300 K and associated energy barriers. (a) Configurational change, reflected by changes in the Te_*i*_–Te distances. The narrow green peak illustrates the shortest Te_*i*_–Te bond, which shows little variation at around 2.8 Å, while the wider pink and orange distributions demonstrate the wide variation in the second and third shortest Te–Te distances (see ESI Fig. S11†). (b) Changes in the orientation of the Te_*i*_–Te bond with respect to the [100] direction. (c) Migration, illustrated by tracking the distance between the current and original position of the interstitial.

To validate these predictions, we perform molecular dynamics under the *NPT* ensemble (300 K, 1 atm, 1 ns), revealing three distinct motions for Te^+1^_*i*_ (Fig. 2). The fastest process corresponds to changes in *configuration* between the *C*_2v_ and *C*_s_ geometries, which is reflected by variations in the distances between Te^+1^_*i*_ and its neighbouring Te atoms as it alternates between forming 1 and 2 Te–Te bonds. On a slower timescale, there are changes in the defect position (*i.e.*, hopping between lattice sites) as well as changes in the *orientation* of the Te–Te bond, indicated by variation in the angle between the Te–Te bond(s) and the lattice axes. These three motions occur rapidly on the nanosecond timescale due to their low energy barriers relative to the thermal energy (*E*_b_ = 28 − 100 meV) with rates on the order of 10^10^ s^−1^ (configurational and hopping) and 10^8^ s^−1^ (rotation) — and highlight the configurational, orientational and migration degrees of freedom that contribute to the defect formation and migration entropies, respectively. In contrast, this dynamic behaviour of Te^+1^_*i*_ differs significantly from that of *V*^+2^_Te_, which remains stable in its *T*_d_ ground state configuration, as depicted in ESI Fig. S12,† and is thus well-described by its static 0 K structure.

### 2.3 Impact of defect entropy on predicted concentrations

The dynamic behaviour of Te^+1^_*i*_ suggests that its formation entropy, *s*_f_, will be significant at the CdTe annealing temperature (≈840 K)^84^ and will thus affect the predicted equilibrium concentration. To verify this, we calculate *s*_f_ and *g*_f_ by considering the different degrees of freedom that change upon forming the defect at a fixed lattice site: electronic, spin, vibrational, orientational and structural (also referred to as configurational).^2^ While the first two terms can be estimated with analytical expressions (see Methods), the vibrational contribution is more challenging and typically requires approximations. By assuming a quadratic energy surface for the interatomic bonds, the harmonic vibrational *g*_f_ can be calculated, which can be extended to account for thermal expansion with the quasiharmonic approximation.^2,86^ However, this harmonic assumption might be limited for defects at high temperatures, where anharmonic effects seem to be important – as suggested by the high anharmonicity scores^87^ observed for Te^+1^_*i*_ relative to pristine CdTe (*σ*(840 K) = 4.5 *versus* 0.8, respectively, and *σ* typically ranging between 0–1; ESI Fig. S13†).

To assess these limitations, we use non-equilibrium thermodynamic integration to account for anharmonic effects and compare the *g*_f_ calculated with each approach. We do this by starting from the Einstein crystal (independent harmonic oscillators), integrating to the anharmonic crystal at 100 K, and finally integrating with respect to temperature up to 840 K (see Methods for further details). Since TI calculates the change in the total free energy described by the MACE potential (*i.e.*, the ionic degrees of freedom), it already includes the vibrational, orientational and structural contributions, and thus we only have to add the electronic and spin terms to *g*^TI^_f_(*T*). We define three defect formation free energies with increasing accuracy:3
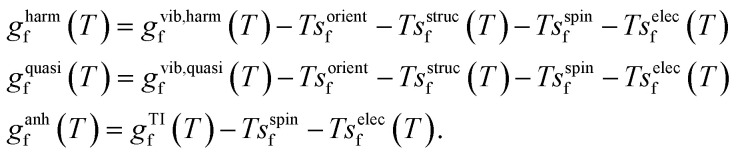
where the (quasi)harmonic vibrational free energy, *g*^vib,harm^_f_, refers to the ground state structure (further details in Methods). Here, the (quasi)harmonic approach decouples all the degrees of freedom, and thus assumes that the timescales for these processes are sufficiently different to avoid significant mixing,^2^ while the anharmonic formalism only decouples the electronic from the ionic motions.

We follow the standard convention in defect chemistry and define *g*_f_ as the change in free energy for forming a defect at a fixed lattice site (*i.e.*, excluding entropic contributions from the mixing or site entropy‡‡This mixing entropy arises from the different ways in which a defect can be arbitrarily placed at the symmetry-equivalent lattice sites and depends on the equilibrium defect concentration. Due to this dependence on *c*, it is separated from the free energy of forming the defect at a fixed site (*g*_f_, which is independent of *c* within the dilute limit) when deriving the expression for the defect concentration.^2^). We calculate the equilibrium defect concentration with4
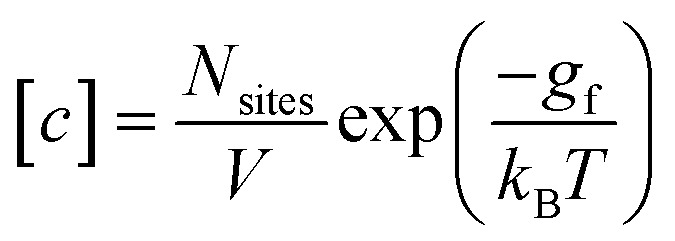
where *V* denotes the crystallographic unit cell volume and *N*_sites_ the number of symmetry-equivalent sites where the defect can form in the unit cell.

**Fig. 3:**
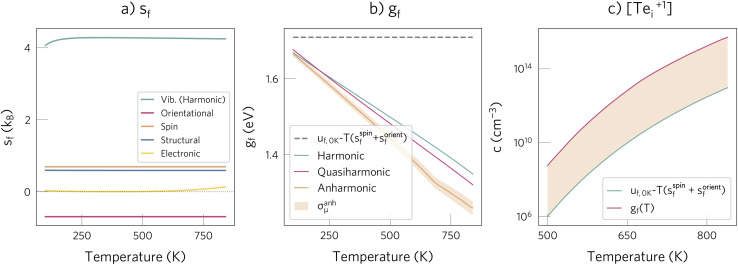
(a) Contribution of the different degrees of freedom to the formation entropy of Te^+1^_*i*_. Note that *s*^orient^_f_ is negative since the symmetry increases when going from the initial to the relaxed interstitial structure (see Methods). (b) Comparison of approximations for calculating the defect formation free energy of Te^+1^_*i*_, *g*_f_(*T*). The shaded orange area illustrates the estimated error in the thermodynamic integration simulations, defined as the standard error of the mean free energy, *σ*^anh^_μ_ (details in Methods). For comparison, the formation internal energy with the spin and orientational entropies, *u*_f_(0 K) − *T*(*s*^spin^_f_ + *s*^orient^_f_), typically used in most defect studies, is shown with a dashed grey line. Note that for Te^+1^_*i*_, *s*^spin^_f_ and *s*^orient^_f_ cancel each other, thus leading to the grey line having zero slope. (c) Effect of including the entropic contribution for predicting the defect concentration.

As demonstrated in Fig. 3b, for Te^+1^_*i*_ thermal effects are significant at annealing temperature, with *g*_f_(840 K) differing by 0.5 eV from *u*_f_(0 K) − *T*(*s*^spin^_f_ + *s*^orient^_f_). All methods are in good agreement, indicating that the harmonic approximation gives a reasonable estimate of *g*_f_, since anharmonic effects approximately cancel out between the bulk and the defect in this temperature range. This agreement also validates the decoupling approximation used to separate the different degrees of freedom (*e.g.*, *g*^TI^_f_ ≈ *g*^vib,harm^_f_ − *Ts*^orient^_f_ − *Ts*^struc^_f_). Using this approximation, we find that their relative entropic contributions follow the expected trend, with the vibrational one dominating, followed by the structural, spin, orientational and electronic terms (with *s*_f_(840 K) of 4.2, 0.7, 0.7, −0.7 and 0.1 *k*_B_, respectively; Fig. 3a). However, we note that the structural term can become larger for defects that have many low-energy metastable configurations.^25^

Overall, the total entropic contribution is not negligible and significantly affects *g*_f_, increasing the predicted concentration by a factor of 500 (Fig. 3c). This importance of entropic effects contrasts with their role in *V*^+2^_Te_, where they are almost negligible (*g*_f_(840 K) − *u*_f_(0 K) = 0.08 eV; ESI Fig. S14†) due to (i) smaller magnitude of the vibrational entropy and (ii) lack of spin, orientational and structural entropies. As a result, we expect thermal effects to be important for defects which (i) introduce strong structural distortions (high *s*^vib^_f_), (ii) break the host site symmetry (high *s*^orient^_f_), and (iii) have low-energy metastable configurations (high *s*^struc^_f_).

## 3. Discussion

Overall, we have illustrated how to model thermal effects for point defects in crystals, demonstrating the dynamic character of Te^+1^_*i*_ in CdTe, which rapidly changes between configuration, orientation and position at room temperature. These degrees of freedom increase the entropy upon defect formation, and can be computed from standard defect calculations as illustrated in this study. The computationally challenging term is the vibrational entropy. We have found that the harmonic approximation gives a reasonable and affordable description of the vibrational formation entropy at 840 K for a dynamic defect with a high anharmonicity score *σ*^anh^. While this suggests the validity of the harmonic approximation for ‘simpler’ defects with single configurations that do not diffuse in this temperature regime, the impact of anharmonicity varies across different defects and host materials, as observed in several metals.^18–20,22,23,26^ This highlights the need for further calculations of *g*^anh^_f_ and *σ*^anh^ on additional defects and host crystals to establish more general guidelines.

By combining the different entropic contributions, we find that thermal effects increase the predicted concentration of Te^+1^_*i*_ by two orders of magnitude, and can thus significantly affect the predicted behaviour by shifting the relative defect populations. Thermal effects will play a significant role for defects that undergo structural reconstructions, break the site symmetry of the host and have low-energy metastable configurations (high *s*^vib^_f_, *s*^orient^_f_ and *s*^struc^_f_), as illustrated by comparing two defects with energy surfaces of different complexity. Beyond defect related factors, hosts with a soft and dynamic lattice or compositional disorder will also be more sensitive to thermal effects, since their defects typically lead to stronger reconstructions (*e.g.*, rebonding or local octahedral rotations in perovskite structures^88^) and display many low-energy metastable configurations (*e.g.*, Sb_2_Se_3_ ^8^ or alloys^89^ like CdSe_*x*_Te_(1−*x*)_^90^). A special case is materials where the phase relevant for applications is only stable at finite temperatures. Here, it can be key to model defects at the device operating temperature since their behaviour can be sensitive to their surrounding structure — as illustrated by the discrepancies when modelling the carrier capture behaviour of I_i_ in different phases of CsPbI_3_.^91^

Despite the importance of including thermal effects for accurate defect predictions, the current limitation is the computational cost. While the orientational, spin and electronic terms can be calculated from standard defect calculations using the doped Python package,^92^ the configurational term requires considering the different thermally-accessible structures of a defect, which can be identified through defect-structure searching methods like ShakeNBreak.^85^ More challenging is the vibrational term as it requires going beyond standard static defect calculations. In practice, accounting for this contribution will only be affordable for high-accuracy studies that target the low-energy defects; especially for applications with a high synthesis or operating temperature, like industrial thermoelectrics, thermochemical water splitting, exhaust automotive catalysts or solid-state fuel cells (*T*_operation_ ≈ 800–1900 K).^93–95^ In these high temperature applications, thermal effects will populate metastable configurations and could also affect the predicted position of the defect charge transition level (*i.e.*, non-negligible entropic term of *T* × [*s*_f_(*q*,*T*) − *s*_f_(*q*′,*T*)]/(*q* − *q*′)) — especially when the change in charge state leads to significant differences in the defect structure and symmetry, spin state and position of the defect level within the bandgap.^80^Finally, based on the performance of our trained models, we note the promise of machine learning force fields for describing the thermal effects of defects. Beyond the prediction of more accurate concentrations, by learning the defect potential energy surface, they can also reduce the cost of modelling defect dynamics at the device operating temperature and on larger time and length scales, which can be key to predict complex processes like defect reactions or diffusion.^96^

## 4. Methodology

### 4.1 Density Functional Theory calculations

All reference calculations were performed with Density Functional Theory using the exchange-correlation functional PBEsol^97^ and the projector augmented wave method,^98^ as implemented in the Vienna *Ab initio* Simulation Package (VASP).^99,100^ We used the standard PAW PBE potentials (version 64) for Te (5s^2^5p^4^) and Cd (4d^10^5s^2^). Although hybrid functionals are typically required to accurately model the electronic behaviour of defects, we used a more affordable GGA functional for several reasons: (i) PBEsol has been found to accurately describe the vibrational properties of crystals;^101^ (ii) it correctly identifies the same defect configurations reported in a previous study using the HSE06 hybrid functional;^12^ and (iii) we aimed to benchmark how to properly train defect MLFFs to reach the high accuracies required to estimate *g*_f_, and as a result needed a functional that would allow a thorough exploration of the configurational landscape up to the CdTe synthesis temperature.

We converged the plane wave energy cutoff and *Γ*-centered *k*-point mesh to 1 meV per atom, resulting in values of 450 eV and 4 × 4 × 4 for the conventional cell of CdTe. To minimise Pulay stress errors during molecular dynamics simulations, we increased the converged energy cutoff by 30% (585 eV). The threshold for electronic convergence was set to 10^−5^ eV.

### 4.2 Training of machine learning force fields

We used the structure similarity kernel implemented in VASP to generate the training sets of configurations using its on-the-fly molecular dynamics approach.^102–105^ This involved heating runs performed under the *NPT* ensemble with a pressure of 1 atm and from an initial temperature of 100 K up to 30% above our target temperature of 840 K. In addition, we generated a series of compressed and expanded structures (0.9–1.1 of the original cell volume) to ensure that the model could be used for the quasiharmonic approximation. For bulk CdTe and its defects, we used a 2 × 2 × 2 supercell of the conventional cell (13.0 Å in length and 64 atoms for bulk CdTe). For Te, we included all the low-energy phases available in the Materials Project^106^ (*E*_hull_ ≤ *k*_B_*T*_synthesis_ = *k*_B_ × (840 K) = 0.08 eV), which were expanded to cubic supercells of at least 10 Å in length. For the liquid Te phase, we generated two models with the packmol code:^107^ two cubic boxes of 15 Å and 17.5 Å in length, containing 95 and 220 atoms, respectively, which gave densities matching the reported values in previous studies at our target temperatures (*ρ* = 0.027 atoms per Å^3^).^108^

An independent model was trained for each system (bulk CdTe, Te^+1^_i_, *V*^+2^_Te_ and Te), with the defect datasets only including one charge state (*e.g.* +1 for Te_i_ and +2 for *V*_Te_). We note that the models for the charged defects were trained on the absolute DFT energies (*e.g.* without electrostatic corrections, which are applied *a posteriori* when calculating the defect formation energy, as described below). Training separate models for each system lead to higher accuracy models than training one *joint* model on the bulk and defect datasets. However, we have observed that training a model *only* on defect configurations should be avoided if the model will be applied to study the absolute energies of larger system sizes (*e.g.*, defect formation energies). While models selectively trained on defect configurations achieve higher accuracy for defective supercells with the same number of atoms, these models lead to a systematic error in the total energies of larger supercells than the supercells used for training, as explained in detail in ESI Section 1C.†

After generating the training sets with VASP, we trained separate MACE^83^ force fields on these datasets to obtain models with higher accuracy and speed. 10% of the configurations in these datasets were used as validation sets to monitor the loss during training. We used a MACE model with Ziegler–Biersack–Littmark (ZBL) pair repulsion,^109^ 2 message passing layers, 256 invariant messages, correlation order of 3, angular resolution of 3 and cutoff radius of 5 Å. The batch size was set to 2 and the Huber loss function was used, with weights of 1, 100 and 100 for the mean square errors in the energies, forces and stresses, respectively. For the last 20% of the training epochs, the weights were updated to values of 1000, 10 and 100 for energy, force and stress, respectively — following the recommended strategy of increasing the weight on the energy errors during the final training epochs. The models were trained until the validation loss converged, which required around 150–200 epochs. The reference energies were defined as the potential energies of isolated Cd and Te atoms.

### 4.3 Validation of machine learning force fields

To generate the test sets, we performed *NPT* molecular dynamics simulations with the trained models at three different temperatures (300, 550 and 900 K), running five independent 24 ps runs at each temperature. We then sampled 100–300 equally-spaced configurations from these trajectories, and performed DFT calculations on them, which were used to calculate the MAE and RMSE on the predicted properties (energy, forces and stresses) of each model (see distribution of sampled configurations and associated errors in Figs. ESI Fig. S1† to ESI Fig. S5†). The MAE and RMSE for the forces and stresses were calculated component wise, as defined in ref. 110. The predicted properties exhibited small absolute errors with no outliers, confirming that the models accurately describe the potential energy surfaces of each system up to 900 K. This extensive testing of the models is especially important for defective crystals, which exhibit more complex energy surfaces than the pristine hosts. In addition, we also validated that the model successfully described the phonons and vibrational free energy of bulk CdTe (see ESI Fig. S6†).

### 4.4 Defect calculations

Defect calculations were setup and analysed using doped.^92^ To account for spurious finite-size supercell effects, the Kumagai–Oba^111^ (eFNV) charge correction scheme was used to calculate *E*_corr_, as automated in doped. The Fermi level was assumed to be located in the middle of the band gap.

### 4.5 Spin degeneracy

The spin degeneracy was calculated with doped using5
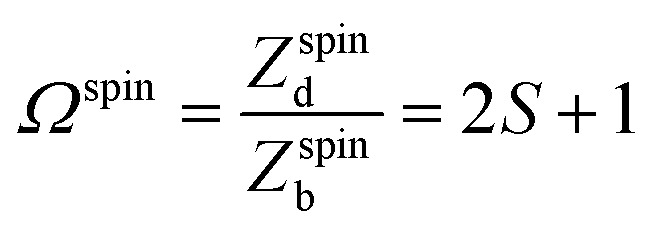
where *Z* denotes the partition function and *S* the total spin angular momentum. For example, Te^+1^_*i*_ has one unpaired electron, resulting in *Ω*^spin^ = 2 × (1/2) + 1 = 2. This degeneracy factor *Ω* can be converted into its respective formation entropy using *s*_f_ = *k*_B_ ln(*Ω*)^2^.

### 4.6 Orientational degeneracy

The orientational degeneracy was also calculated with doped using6
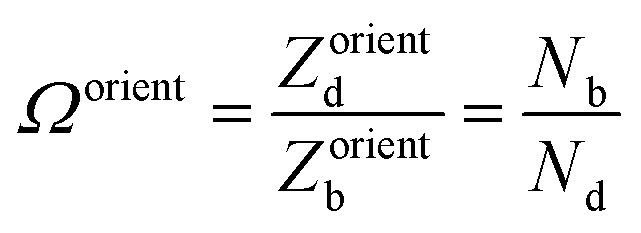
where *N* is the number of symmetry operations of the defect site in the bulk (b) and defective (d) supercells.^2^ As discussed in the doped documentation,^92^ for vacancies and substitutions there is a clear definition of the defect site in the pristine supercell (*e.g.*, the lattice site where the vacancy/substitution forms). In contrast, for interstitials, the definition of the lattice site in the bulk can be ambiguous, which affects the *partition* between orientational degeneracy and site multiplicity. Here, we follow the definition adopted by Kavanagh *et al.*^92^ where the interstitial site in the bulk is defined as the relaxed site of the interstitial but with all other atoms fixed in their bulk (unrelaxed) positions, while the interstitial site in the *defect* supercell corresponds to the relaxed position of *both* the interstitial and all other atoms. Accordingly, the site multiplicity is determined for the lattice site that the interstitial occupies after relaxation and with all other atoms fixed in their bulk positions. Note that other definitions can be adopted and will lead to the same total prefactor (*Ω*^orient^ × *N*_site_) but different partitions into the orientational and site degeneracies.

For Te^+1^_*i*_, the *C*_2v_ configuration has an initial site symmetry of *C*_s_ which becomes *C*_2v_ when the atoms around the interstitial relax due to the formation of the Te–Te dimer. Similarly, the (metastable) *C*_s_ configuration has an initial *C*_1_ site symmetry which becomes *C*_s_ after the relaxation. As a result, for both configurations the orientational degeneracy *Ω*^orient^ is 0.5 (*e.g.* the site symmetry increases upon relaxation of the atoms around the interstitial). The site multiplicities per primitive cell are 12 and 24 for the *C*_2v_ and *C*_s_ configurations, respectively. These degeneracies are accounted for when predicting the defect concentration (eqn (4)) where the orientational entropy is included in *g*_f_.

### 4.7 Electronic entropy

The electronic entropy was calculated using the fixed density of states (DOS) approximation,^56,112–115^ that assumes a temperature-independent DOS. Since the electronic entropy is sensitive to the bandgap, and PBEsol significantly underestimates it, we performed self-consistent field HSE06 (*α* = 0.345 (ref. 13)) calculations on the 0 K structures optimised with PBEsol. The electronic entropy is then calculated using7
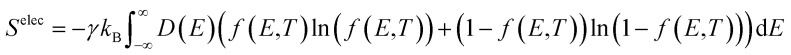
where *γ* equals 1 for spin-polarized systems and 2 for spin-unpolarized systems.^113^*D*(*E*) is the electronic density of states at energy *E* (calculated at 0 K) and *f*(*E*) is the occupation of the energy level *E* given by Fermi–Dirac occupation statistics8
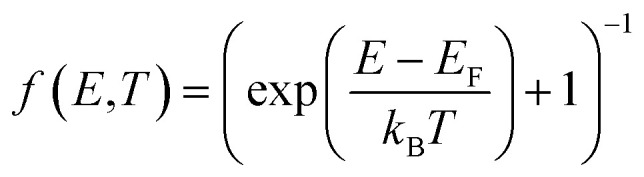
with *E*_F_ denoting the Fermi level. We define the formation electronic entropy as the entropy change in the reaction (CdTe)_32_ + Te → Cd_32_Te^+1^_33_ + e^−^ (ESI Fig. S17†), with *E*_F_ for each of these terms calculated as follows:

• Te, CdTe and Cd_32_Te^+1^_33_: The Fermi level is assumed to be located mid-gap between the highest occupied and lowest unoccupied state. Note that, in theory, the Fermi level of CdTe should correspond to the *self-consistent* value determined for a set of defects and charge states.^116,117^ Determining *E*_F_ would require calculating the formation energies for all possible intrinsic defects in CdTe, which is beyond the scope of our study. Accordingly, we assume the Fermi level to be midgap.^38^ Te^+1^_*i*_ introduces an empty state 0.75 eV above the valence band maximum (VBM), and thus *E*_F_ is set to 0.75/2 = 0.37 eV above the VBM. Note that the electronic entropy is sensitive to the energy difference between *E*_F_ and the lowest unoccupied state, thus requiring an accurate electronic structure.

• Extra electron: It is defined as the excess electronic entropy when one extra electron is added to the conduction band minimum (CBM) of the bulk system, *i.e. s*^elec^(*N* + 1) − *s*^elec^(*N*), where *s*^elec^(*N*) denotes the electronic entropy of bulk CdTe with *N* and *N* + 1 electrons. To calculate the electronic entropy of the *N* + 1 system, we determine *E*_F_ with^118^9
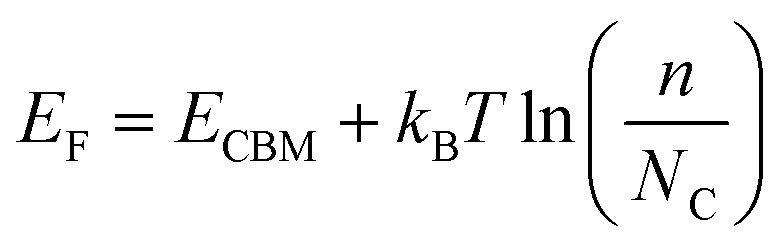
where *N*_C_ denotes the effective density of states in the conduction band, given by10
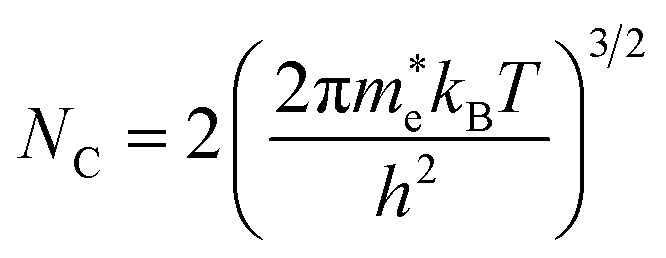
with 
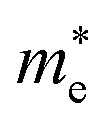
 and *h* representing the electron effective mass and Planck constant, respectively. For the concentration of excess electrons donated by the defect, *n*, we assume a value of *n* = 10^15^ cm^−3^, which equals our predicted value for the equilibrium concentration of Te^+1^_*i*_ at 840 K (thus making the reaction (CdTe)_32_ + Te → Cd_32_Te^+1^_33_ + e^−^ charge neutral). We note that if all possible point defects were considered, the value of *n* should be set to the excess electron concentration, as determined self-consistently.^116^

Finally, we note that the electronic entropy can become significant at elevated temperatures (*T* ≥ 1000 K, ESI Fig. S17†) if the defect i) introduces an (occupied) empty state close to the (CBM) VBM or ii) changes the occupation of localised d/f bands of nearby cations (*e.g.*, *V*^+2^_O_ reducing two Ce^+4^ to Ce^+3^ in CeO_2_), as demonstrated in previous studies.^93,119^

### 4.8 Structural entropy

The Python code ShakeNBreak was used to identify the defect ground state and metastable configurations.^4,5,85^ From these configurations, the structural or configurational entropy can be estimated using11
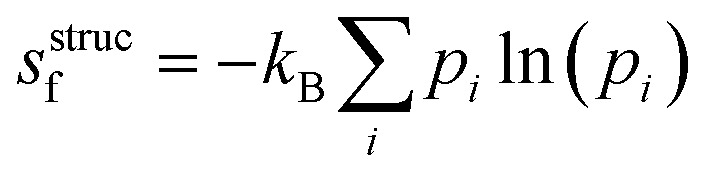
where *p*_*i*_ denotes the Boltzmann probability of configuration *i*, given by12
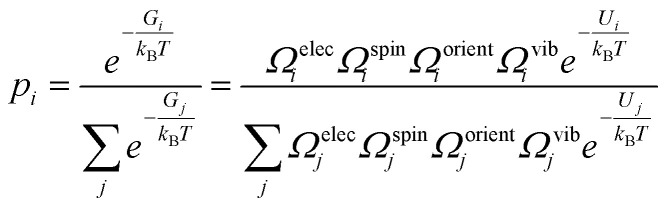
where *G*_*i*_ and *U*_*i*_ are the Gibbs free energy and internal energy of configuration *i*, and we have included the degeneracy factors *Ω* since these can be configuration dependent. In practice, the main degrees of freedom that change between configurations are the orientational, spin and vibrational, thus simplifying eqn (12) to13
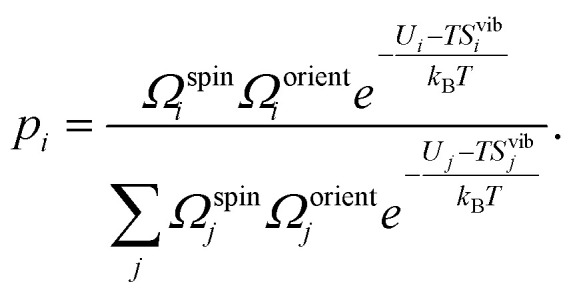


Beyond applying this analytical approach, we also calculated the structural entropy using the ‘inherent structures’ method (IS).^25^ Within this formalism, we performed *NPT* MD trajectories at 30 temperatures (equally spaced ranging from 100 to 840 K), then sampled 1600 equally-spaced configurations and performed conjugate gradient optimisations to quench the structures to the nearest local minima in the 0 K PES (ESI Fig. S16†). The configurational entropy was then calculated with14
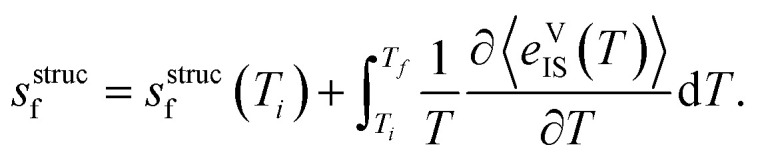
where 
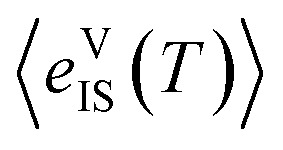
 denotes the average potential energy of the inherent structures sampled at *T*, with the volume fixed to the optimal value for the 0 K ground state structure, and *T*_*i*_ and *T*_f_ set to 100 and 840 K, respectively. This method resulted in *s*^struc^_f_(840 K) = 1.05 *k*_B_, in the same order of the value of 0.6 *k*_B_ obtained with the analytical method. The slightly larger value of *s*^struc^_f_ estimated with the IS formalism likely stems from the additional intermediate configurations sampled with quenching.

Finally, a third approach to account for the structural entropy involves coarse-graining the configurational degree of freedom, and calculating the total defect concentration as a sum over the different configurations *i* using^2^15
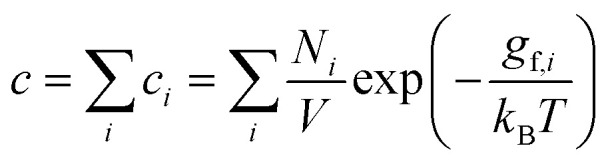
where *N*_*i*_ and *g*_f,*i*_ denote the number of symmetry-equivalent sites and formation free energy of configuration *i* (with *g*_f,*i*_ = *u*_f,*i*_ − *T*(*s*^vib,harm^_f,*i*_ + *s*^orient^_f,*i*_ + *s*^spin^_f,*i*_ + *s*^elec^_f,*i*_)). From this expression, an effective formation free energy can be obtained by16
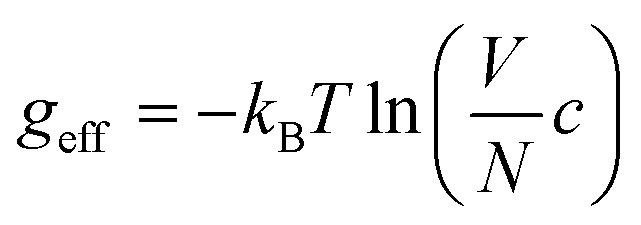
where *N* denotes the number of symmetry-equivalent sites for the ground state structure. By comparing *g*_eff_ with the *g*_f_ calculated using eqn (11), we verified that these values agree (see ESI Fig. S18†).

### 4.9 Vibrational entropy

The harmonic and quasiharmonic vibrational free energies were calculated using phonopy.^86,120^ Within the quasiharmonic framework, which includes the effect of thermal expansion on the phonon frequencies, we generated 11 structures by scaling the supercell volume by factors ranging from 0.9 to 1.10 with a 0.02 increment.

### 4.10 Thermodynamic integration

Fully anharmonic free energies were calculated using non-equilibrium TI in LAMMPS,^121,122^ as implemented in the code calphy.^81^ This involved two thermodynamic integration paths: first, we integrate from the Einstein crystal to the anharmonic one at 100 K (Frenkel Ladd method) and then we calculate the temperature variation of the free energy at constant pressure by integrating from 100 K to 840 K (commonly known as reversible scaling). These simulations were performed fixing the center of mass, as done in previous studies.^18,81,122^ For each path, we performed 10 independent TI runs to estimate the error, defined as the standard error of the mean free energy (
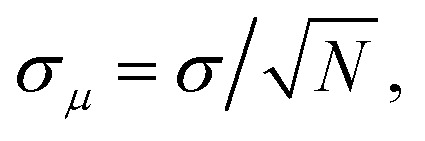
, where *σ* denotes the standard deviation in the individual free energies calculated from the *N* = 10 independent TI runs). The switching time was adjusted for each system until the error converged to an acceptable value (see ESI Fig. S15†), which must be very low (*σ*_μ_ < 0.25 meV per atom ≈ 20 meV per supercell) to get an accurate *g*_f_. Since *g*_f_ is the difference between two large and similar numbers (the free energies of the bulk and defect supercells), small relative errors in either of these quantities can lead to a large error in *g*_f_. The convergence tests resulted in the switching times reported in Table 2.

**Table 2:** Equilibration and switching times in picoseconds used for the thermodynamic integration paths of each system involved in the defect formation reaction. The timestep was set to 2 fs

Path	CdTe	Te^+1^_*i*_	V^+2^_Te_	Te
Einstein → anharmonic	25, 100	25, 200	15, 150	25, 70
100 K → 840 K	25, 170	25, 1000	25, 1000	25, 70
840 K → 500 K	—	—	—	25, 70

We note that during the temperature scaling runs of the interstitial, defect diffusion occurs within the simulation timescale. This migration, which arises from the shape of the potential energy surface, contributes to the anharmonic free energy through the sampling of intermediate structures during site hopping. We expect that their contribution is small as the defects spend more time around their local minima configurations.

Finally, calculating the temperature dependence of the free energy for tellurium is slightly more challenging since it melts at 722 K.^123^ Accordingly, we performed two TI simulations:^81^ (i) Einstein crystal (100 K) → anharmonic crystal (100 K) → anharmonic crystal (840 K) and (ii) Uhlenbeck–Ford model (840 K) → liquid Te (840 K) → liquid Te (500 K). By comparing the free energies from both simulations, we determined the phase transition temperature and the variation of the free energy with temperature (ESI Fig. S19†). The calculated phase transition temperature is 704 K, which is in good agreement with the experimentally reported value of 722 K.^123^

### 4.11 Molecular dynamics

To model the behaviour of the defects at room temperature, we performed *NPT* molecular dynamics with LAMMPS^121^ using both a 65-atom (*a* = 13 Å) and 513-atom cubic supercells (*a* = 26 Å) to properly capture the dynamics and diffusion of the interstitial. The Nosé–Hoover thermostat and barostat were used (1 atm, 300 K and timestep of 2 fs) with equilibration and production times of 300 ps and 1 ns, respectively. These trajectories were analysed with Python using tools from the ase,^124^ pymatgen-analysis-defects,^125,126^ pymatgen,^127–129^ dscribe,^130,131^ umap,^132^ direct,^133^ matplotlib,^134^ and seaborn^135^ packages, and visualised with Ovito^136^ and CrystalMaker.^137^

The energy barriers for the changes in configuration, orientation and position of Te^+1^_*i*_ were calculated with the Nudged Elastic Band method,^138,139^ as implemented in ase.^124^ The associated rates for these processes were calculated using Transition State Theory (*e.g. k*(*T*) = *ν* exp(−*E*_b_/(*k*_B_*T*)) and approximating the attempt frequency *ν* by the curvature of the PES at the initial state. The reaction pathways between defect configurations were represented in one dimension using a generalised mass-weighted displacement coordinate *Q*, calculated using17
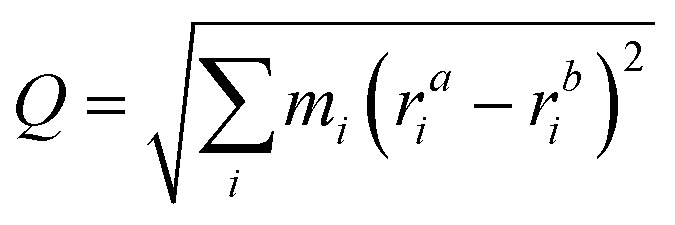
where *m*_*i*_ and *r*_*i*_ represent the mass and atomic position of atom *i* in two configurations *a* and *b*. The anharmonicity scores were calculated with FHI-vibes^87,140^ on MD trajectories (*NPT* ensemble; 1 atm, 500 ps) at three temperatures (300, 550 and 900 K) for pristine CdTe and Te^+1^_i_.

## Data availability

The datasets and trained models are available from the Zenodo repository https://zenodo.org/records/15224961 (DOI: https://doi.org/10.1039/d4sc08582e).

## Author contributions

Conceptualisation & project administration: A. W., I. M.-L. Investigation, methodology and formal analysis: I. M.-L. Supervision: A. W. Writing – original draft: I. M.-L. Writing – review & editing: all authors. Resources and funding acquisition: A. W. These author contributions are defined according to the CRediT contributor roles taxonomy.

## Conflicts of interest

The authors declare no competing interests.

## Supplementary Material

SC-016-D4SC08582E-s001

## References

[cit1] Stoneham A. M. , Theory of defects in solids, Oxford University Press, 1975

[cit2] Mosquera-Lois I., Kavanagh S. R., Klarbring J., Tolborg K., Walsh A. (2023). Chem. Soc. Rev..

[cit3] Freysoldt C., Grabowski B., Hickel T., Neugebauer J., Kresse G., Janotti A., Van De Walle C. G. (2014). Rev. Mod. Phys..

[cit4] Mosquera-Lois I., Kavanagh S. R., Walsh A., Scanlon D. O. (2023). npj Comput. Mater..

[cit5] Mosquera-Lois I., Kavanagh S. R. (2021). Matter.

[cit6] Wang X., Kavanagh S. R., Scanlon D. O., Walsh A. (2023). Phys. Rev. B.

[cit7] Wang X., Kavanagh S. R., Walsh A. (2025). ACS Energy Lett..

[cit8] Wang X., Kavanagh S. R., Scanlon D. O., Walsh A. (2024). Joule.

[cit9] Squires A. G., Ganeshkumar L., Savory C. N., Kavanagh S. R., Scanlon D. O. (2024). ACS Energy Lett..

[cit10] Yang J.-H., Shi L., Wang L.-W., Wei S.-H. (2016). Sci. Rep..

[cit11] Alkauskas A., Dreyer C. E., Lyons J. L., Van de Walle C. G. (2016). Phys. Rev. B.

[cit12] Kavanagh S. R., Scanlon D. O., Walsh A., Freysoldt C. (2022). Faraday Discuss..

[cit13] Kavanagh S. R., Walsh A., Scanlon D. O. (2021). ACS Energy Lett..

[cit14] Kavanagh S. R., Nielsen R. S., Hansen J. L., Davidsen R. S., Hansen O., Samli A. E., Vesborg P. C., Scanlon D. O., Walsh A. (2025). Energy Environ. Sci..

[cit15] Guo D., Qiu C., Yang K., Deng H.-X. (2021). Phys. Rev. Appl..

[cit16] Fowler W. B., Stavola M., Venzie A., Portoff A. (2024). J. Appl. Phys..

[cit17] Bochkarev A. S., van Roekeghem A., Mossa S., Mingo N. (2019). Phys. Rev. Mater..

[cit18] Cheng B., Ceriotti M. (2018). Phys. Rev. B.

[cit19] Chiesa S., Derlet P. M., Dudarev S. L. (2009). Phys. Rev. B.

[cit20] de Koning M., Miranda C. R., Antonelli A. (2002). Phys. Rev. B.

[cit21] De Koning M., Ramos De Debiaggi S., Monti A. (2003). Defect Diffus. Forum.

[cit22] Glensk A., Grabowski B., Hickel T., Neugebauer J. (2014). Phys. Rev. X.

[cit23] Grabowski B., Ismer L., Hickel T., Neugebauer J. (2009). Phys. Rev. B.

[cit24] Lucas G., Schäublin R. (2009). Nucl. Instrum. Methods Phys. Res..

[cit25] Luo J., Zhou C., Cheng Y., Li Q., Liu L., Douglas J. F., Sinno T. (2022). Phys. Rev. Mater..

[cit26] Mellan T. A., Duff A. I., Grabowski B., Finnis M. W. (2019). Phys. Rev. B.

[cit27] Safonova E. V., Mitrofanov Y. P., Konchakov R. A., Vinogradov A. Y., Kobelev N. P., Khonik V. A. (2016). J. Phys.: Condens. Matter.

[cit28] Satta A., Willaime F., de Gironcoli S. (1998). Phys. Rev. B.

[cit29] Smirnov G. S., Stegailov V. V. (2019). J. Phys.: Condens. Matter.

[cit30] Shin D., Wolverton C. (2012). Acta Mater..

[cit31] Gong Y., Grabowski B., Glensk A., Körmann F., Neugebauer J., Reed R. C. (2018). Phys. Rev. B.

[cit32] Smirnova D., Starikov S., Leines G. D., Liang Y., Wang N., Popov M. N., Abrikosov I. A., Sangiovanni D. G., Drautz R., Mrovec M. (2020). Phys. Rev. Mater..

[cit33] Zhang X., Grabowski B., Hickel T., Neugebauer J. (2018). Comput. Mater. Sci..

[cit34] Mathes L., Gigl T., Leitner M., Hugenschmidt C. (2020). Phys. Rev. B.

[cit35] Sinno T., Jiang Z. K., Brown R. A. (1996). Appl. Phys. Lett..

[cit36] Al-Mushadani O. K., Needs R. J. (2003). Phys. Rev. B.

[cit37] Rauls E., Frauenheim T. (2004). Phys. Rev. B.

[cit38] Blöchl P. E., Smargiassi E., Car R., Laks D. B., Andreoni W., Pantelides S. T. (1993). Phys. Rev. Lett..

[cit39] Maroudas D., Brown R. A. (1993). Phys. Rev. B.

[cit40] Mendelev M. I., Mishin Y. (2009). Phys. Rev. B.

[cit41] Ungar P. J., Halicioglu T., Tiller W. A. (1994). Phys. Rev. B.

[cit42] Wynblatt P. (1969). J. Phys. Chem. Solids.

[cit43] Harding J. H. (1985). Physica B+C.

[cit44] Harding J. H., Stoneham A. M. (1981). Philos. Mag. B.

[cit45] Harding J. H. (1985). Phys. Rev. B.

[cit46] Luo J., Zhou C., Li Q., Liu L. (2022). J. Chem. Phys..

[cit47] Luo J., Zhou C., Li Q., Liu L. (2022). Materials.

[cit48] Mishin Y., Sorensen M. R., Voter A. F. (2001). Philos. Mag. A.

[cit49] Wynblatt P. (1969). Phys. Status Solidi B.

[cit50] Lapointe C., Swinburne T. D., Proville L., Becquart C. S., Mousseau N., Marinica M.-C. (2022). Phys. Rev. Mater..

[cit51] Jacobs P. W. M. (1990). J. Chem. Soc., Faraday Trans..

[cit52] Nam J. and Gómez-Bombarelli R., arXiv, 2024, preprint, arXiv:2404.10746, 10.48550/arXiv.2404.10746

[cit53] Ramos de Debiaggi S., de Koning M., Monti A. M. (2006). Phys. Rev. B.

[cit54] Carling K. M., Wahnström G., Mattsson T. R., Sandberg N., Grimvall G. (2003). Phys. Rev. B.

[cit55] Foiles S. M. (1994). Phys. Rev. B.

[cit56] Estreicher S. K., Sanati M., West D., Ruymgaart F. (2004). Phys. Rev. B.

[cit57] Sanati M., Estreicher S. K. (2003). Phys. B.

[cit58] Sanati M., Estreicher S. K. (2003). Solid State Commun..

[cit59] Sanati M., Estreicher S. K. (2005). Phys. Rev. B.

[cit60] Smargiassi E., Car R. (1996). Phys. Rev. B.

[cit61] Catlow C., Corish J., Jacobs P., Lidiard A. (1981). J. Phys. C:Solid State Phys..

[cit62] Ágoston P., Albe K. (2009). Phys. Chem. Chem. Phys..

[cit63] Zacherle T., Schmidt P. C., Martin M. (2013). Phys. Rev. B.

[cit64] Smith T., Moxon S., Tse J. S., Skelton J. M., Cooke D. J., Gillie L. J., Silva E. L. d., Harker R. M., Storr M. T., Parker S. C., Molinari M. (2023). J. Phys.: Energy.

[cit65] Zacherle T., Schriever A., De Souza R. A., Martin M. (2013). Phys. Rev. B.

[cit66] Walsh A., Sokol A. A., Catlow C. R. A. (2011). Phys. Rev. B:Condens. Matter Mater. Phys..

[cit67] Baldassarri B., He J., Wolverton C. (2024). Phys. Rev. Mater..

[cit68] Millican S. L., Clary J. M., Musgrave C. B., Lany S. (2022). Chem. Mater..

[cit69] Moxon S., Skelton J., Tse J. S., Flitcroft J., Togo A., Cooke D. J., Silva E. L. d., Harker R. M., Storr M. T., Parker S. C., Molinari M. (2022). J. Mater. Chem. A.

[cit70] Sun Y., Liu T., Chang Q., Ma C. (2018). J. Phys. Chem. Solids.

[cit71] Miceli G., Pasquarello A. (2016). Phys. Rev. B.

[cit72] Grieshammer S., Zacherle T., Martin M. (2013). Phys. Chem. Chem. Phys..

[cit73] Cazorla C. (2017). Phys. Rev. Appl..

[cit74] Wynn J. M. , Needs R. J. and Morris A. J., arXiv, 2016, preprint, arXiv:1609.04760, 10.48550/arXiv.1609.04760

[cit75] Youssef M., Yildiz B. (2012). Phys. Rev. B.

[cit76] Holtzman L. N., Vargas P. A., Hennig R. G., Barmak K. (2024). J. Chem. Phys..

[cit77] Gorfer A., Abart R., Dellago C. (2024). Phys. Rev. Mater..

[cit78] Tarento R. J., Harding J. H. (1987). J. Phys. C: Solid State Phys..

[cit79] Zhang C., Gygi F., Galli G. (2024). Phys. Rev. Mater..

[cit80] Bjørheim T. S., Arrigoni M., Gryaznov D., Kotomin E., Maier J. (2015). Phys. Chem. Chem. Phys..

[cit81] Menon S., Lysogorskiy Y., Rogal J., Drautz R. (2021). Phys. Rev. Mater..

[cit82] Kapil V., Engel E., Rossi M., Ceriotti M. (2019). J. Chem. Theory Comput..

[cit83] Batatia I., Kovacs D. P., Simm G., Ortner C., Csanyi G. (2022). Adv. Neural Inf. Process. Syst..

[cit84] Metzger W. K., Grover S., Lu D., Colegrove E., Moseley J., Perkins C. L., Li X., Mallick R., Zhang W., Malik R., Kephart J., Jiang C.-S., Kuciauskas D., Albin D. S., Al-Jassim M. M., Xiong G., Gloeckler M. (2019). Nat. Energy.

[cit85] Mosquera-Lois I., Kavanagh S. R., Walsh A., Scanlon D. O. (2022). J. Open Source Softw..

[cit86] Togo A., Chaput L., Tadano T., Tanaka I. (2023). J. Phys.:Condens. Matter.

[cit87] Knoop F., Purcell T. A. R., Scheffler M., Carbogno C. (2020). Phys. Rev. Mater..

[cit88] Choi M., Oba F., Kumagai Y., Tanaka I. (2013). Adv. Mater..

[cit89] Park J., Xu B., Pan J., Zhang D., Lany S., Liu X., Luo J., Qi Y. (2023). npj Comput. Mater..

[cit90] Mosquera-Lois I., Kavanagh S. R., Ganose A. M., Walsh A. (2024). npj Comput. Mater..

[cit91] Whalley L. D. (2023). J. Phys. Chem. C.

[cit92] Kavanagh S. R., Squires A. G., Nicolson A., Mosquera-Lois I., Ganose A. M., Zhu B., Brlec K., Walsh A., Scanlon D. O. (2024). J. Open Source Softw..

[cit93] Naghavi S. S., Emery A. A., Hansen H. A., Zhou F., Ozolins V., Wolverton C. (2017). Nat. Commun..

[cit94] Sikstrom D., Thangadurai V. (2024). Ionics.

[cit95] Gao J., Tian G., Sorniotti A., Karci A. E., Di Palo R. (2019). Appl. Therm. Eng..

[cit96] Zhong X., Höfling F., John T. (2025). Geochem., Geophys., Geosyst..

[cit97] Perdew J. P., Ruzsinszky A., Csonka G. I., Vydrov O. A., Scuseria G. E., Constantin L.
A., Zhou X., Burke K. (2008). Phys. Rev. Lett..

[cit98] Kresse G., Furthmüller J. (1996). Comput. Mater. Sci..

[cit99] Kresse G., Hafner J. (1993). Phys. Rev. B.

[cit100] Kresse G., Hafner J. (1994). Phys. Rev. B.

[cit101] Skelton J. M., Tiana D., Parker S. C., Togo A., Tanaka I., Walsh A. (2015). J. Chem. Phys..

[cit102] Jinnouchi R., Lahnsteiner J., Karsai F., Kresse G., Bokdam M. (2019). Phys. Rev. Lett..

[cit103] Jinnouchi R., Karsai F., Kresse G. (2019). Phys. Rev. B.

[cit104] Jinnouchi R., Miwa K., Karsai F., Kresse G., Asahi R. (2020). J. Phys. Chem. Lett..

[cit105] Liu P., Verdi C., Karsai F., Kresse G. (2022). Phys. Rev. B.

[cit106] Jain A., Ong S. P., Hautier G., Chen W., Richards W. D., Dacek S., Cholia S., Gunter D., Skinner D., Ceder G., Persson K. A. (2013). APL Mater..

[cit107] Martínez L., Andrade R., Birgin E. G., Martínez J. M. (2009). J. Comput. Chem..

[cit108] Akola J., Jones R. O., Kohara S., Usuki T., Bychkov E. (2010). Phys. Rev. B.

[cit109] Ziegler J. F. and Biersack J. P., in The Stopping and Range of Ions in Matter, ed. D. A. Bromley, Springer US, Boston, MA, 1985, pp. 93–129

[cit110] Morrow J. D., Gardner J. L. A., Deringer V. L. (2023). J. Chem. Phys..

[cit111] Kumagai Y., Oba F. (2014). Phys. Rev. B:Condens. Matter Mater. Phys..

[cit112] Eriksson O., Wills J. M., Wallace D. (1992). Phys. Rev. B.

[cit113] Zhang X., Grabowski B., Körmann F., Freysoldt C., Neugebauer J. (2017). Phys. Rev. B.

[cit114] Metsue A., Oudriss A., Bouhattate J., Feaugas X. (2014). J. Chem. Phys..

[cit115] Willaime F., Satta A., Nastar M., Le Bacq O. (2000). Int. J. Quantum Chem..

[cit116] Squires A. G., Scanlon D. O., Morgan B. J. (2023). J. Open Source Softw..

[cit117] Buckeridge J. (2019). Comput. Phys. Commun..

[cit118] Smets A. H. M. , Jäger K., Isabella O., Swaaij R. A. v. and Zeman M., Solar energy : the physics and engineering of photovoltaic conversion, technologies and systems, UIT Cambridge Ltd, Cambridge, England, 2016

[cit119] Lany S. (2018). J. Chem. Phys..

[cit120] Togo A., Tanaka I. (2015). Scr. Mater..

[cit121] Thompson A. P., Aktulga H. M., Berger R., Bolintineanu D. S., Brown W. M., Crozier P. S., in ’t Veld P. J., Kohlmeyer A., Moore S. G., Nguyen T. D., Shan R., Stevens M. J., Tranchida J., Trott C., Plimpton S. J. (2022). Comput. Phys. Commun..

[cit122] Freitas R., Asta M., de Koning M. (2016). Comput. Mater. Sci..

[cit123] Kracek F. C. (1941). J. Am. Chem. Soc..

[cit124] Larsen A. H., Mortensen J. J., Blomqvist J., Castelli I. E., Christensen R., Dułak M., Friis J., Groves M. N., Hammer B., Hargus C., Hermes E. D., Jennings P. C., Jensen P. B., Kermode J., Kitchin J. R., Kolsbjerg E. L., Kubal J., Kaasbjerg K., Lysgaard S., Maronsson J. B., Maxson T., Olsen T., Pastewka L., Peterson A., Rostgaard C., Schiøtz J., Schütt O., Strange M., Thygesen K. S., Vegge T., Vilhelmsen L., Walter M., Zeng Z., Jacobsen K. W. (2017). World J. Condens. Matter Phys..

[cit125] Shen J.-X., Varley J. (2024). J. Open Source Softw..

[cit126] Shen J.-X., Voss L. F., Varley J. B. (2024). J. Appl. Phys..

[cit127] Ong S. P., Richards W. D., Jain A., Hautier G., Kocher M., Cholia S., Gunter D., Chevrier V. L., Persson K. A., Ceder G. (2013). Comput. Mater. Sci..

[cit128] Jain A., Ong S. P., Hautier G., Chen W., Richards W. D., Dacek S., Cholia S., Gunter D., Skinner D., Ceder G., Persson K. A. (2013). APL Mater..

[cit129] Ong S. P., Cholia S., Jain A., Brafman M., Gunter D., Ceder G., Persson K. A. (2015). Comput. Mater. Sci..

[cit130] Himanen L., Jäger M. O. J., Morooka E. V., Federici Canova F., Ranawat Y. S., Gao D. Z., Rinke P., Foster A. S. (2020). Comput. Phys. Commun..

[cit131] Laakso J., Himanen L., Homm H., Morooka E. V., Jäger M. O., Todorović M., Rinke P. (2023). J. Chem. Phys..

[cit132] McInnes L., Healy J., Saul N., Großberger L. (2018). J. Open Source Softw..

[cit133] Qi J., Ko T. W., Wood B. C., Pham T. A., Ong S. P. (2024). npj Comput. Mater..

[cit134] Hunter J. D. (2007). Comput. Sci. Eng..

[cit135] Waskom M. L. (2021). J. Open Source Softw..

[cit136] Stukowski A. (2010). Modell. Simul. Mater. Sci. Eng..

[cit137] CrystalMaker®: a crystal and molecular structures program for Mac and Windows. CrystalMaker Software Ltd, Oxford, England (https://www.crystalmaker.com

[cit138] Henkelman G., Uberuaga B. P., Jónsson H. (2000). J. Chem. Phys..

[cit139] Henkelman G., Jónsson H. (2000). J. Chem. Phys..

[cit140] Knoop F., Purcell T. A. R., Scheffler M., Carbogno C. (2020). J. Open Source Softw..

