# Supplementary material for: Point defect formation at finite temperatures with machine learning force fields
Source: Chem Sci. 2025 Apr 8;16(20):8878–88. doi: 10.1039/d4sc08582e (PMC12012633; doi:10.1039/d4sc08582e)
Supplement: SC-016-D4SC08582E-s001 [file SC-016-D4SC08582E-s001.pdf]

Supplementary Information (SI) for Chemical Science.
This journal is © The Royal Society of Chemistry 2025

Supplementary Information for 'Point defect formation at finite temperatures with machine learning force fields'

1. MACHINE LEARNING FORCE FIELDS

A. Validation on test set

We validated the accuracy of our machine learning force fields by comparing their predicted energies, forces and stresses on a test set of configurations with the ground-truth DFT values. As shown in Supplementary Figure S1 to Supplementary Figure S4, the predicted properties exhibit small absolute errors with no outliers, demonstrating the the models accurately describe the potential energy surfaces of each system up to 900 K. Carefully analysing the distribution of the errors is key to assess the accuracy of the models for the defective systems, which have more complex energy landscapes than pristine crystals and as a result are more challenging to describe. An especially challenging case would involve a defect that exhibits both deep (localised) and shallow (band-like/delocalised) states for a single charge state. While we did not observe this behaviour in $\mathrm{Te}_\mathrm{i}^{+1}$ or ${V_\mathrm{Te}}^{+2}$, it should be taken into account when training defect models.

Supplementary Table S1 Number of configurations in the datasets used to train and test each model. The training datasets are divided into training and validation sets (90% and 10%, respectively), with the latter used to monitor the validation loss during training. We note that we used more training configurations than necessary, as discussed in the 1 B.

	CdTe	$\mathrm{Te}_\mathrm{i}^{+1}$	${V_\mathrm{Te}}^{+2}$	Te
Train	1412	3992	4316	1171
Test	300	237	132	312

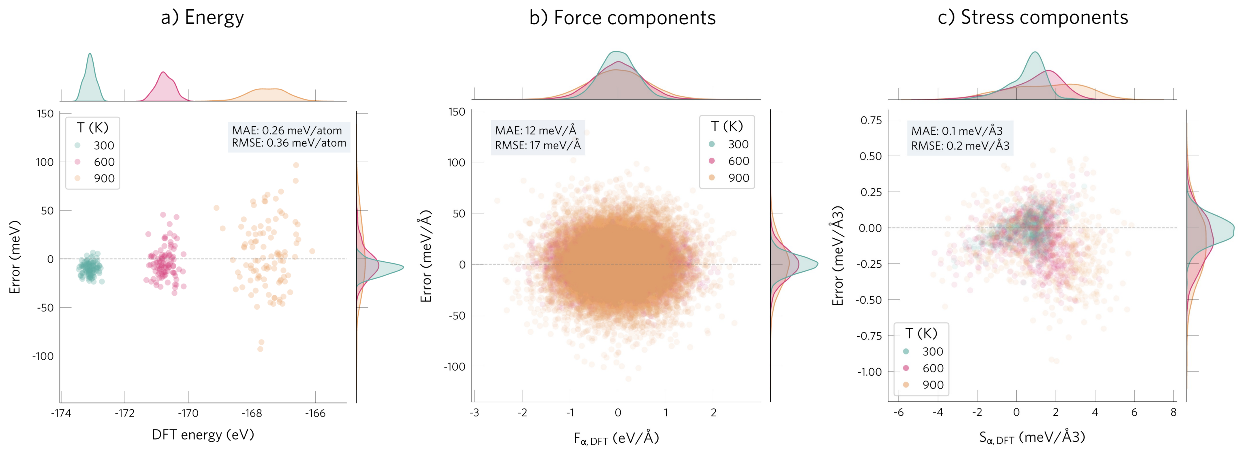

Supplementary Figure S1 Distribution of mean absolute and root mean squared errors (MAE, RMSE) for the test set of bulk CdTe.

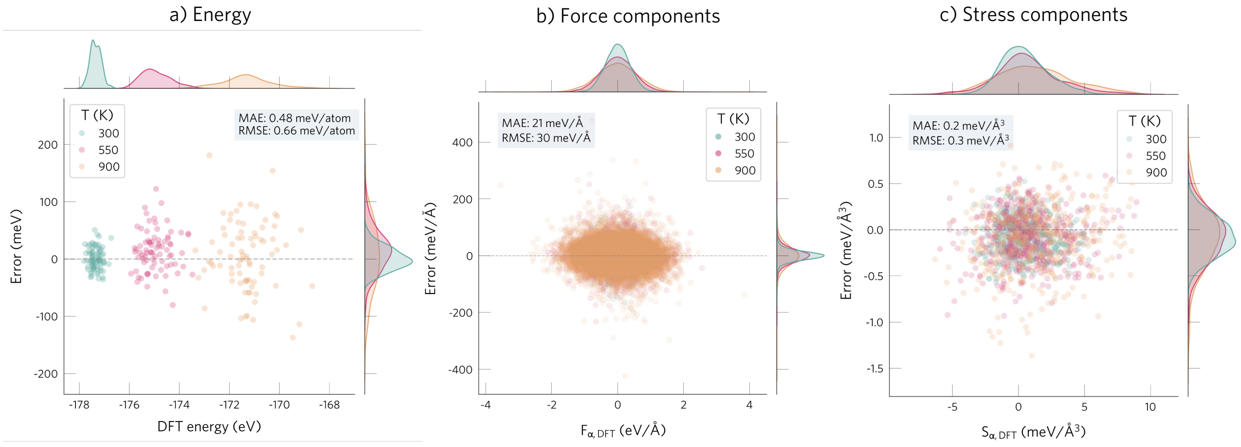

Supplementary Figure S2 Distribution of mean absolute and root mean squared errors (MAE, RMSE) for the test set of Te_i^{+1}. The distribution of the test and training configurations are illustrated in Supplementary Figure S5.

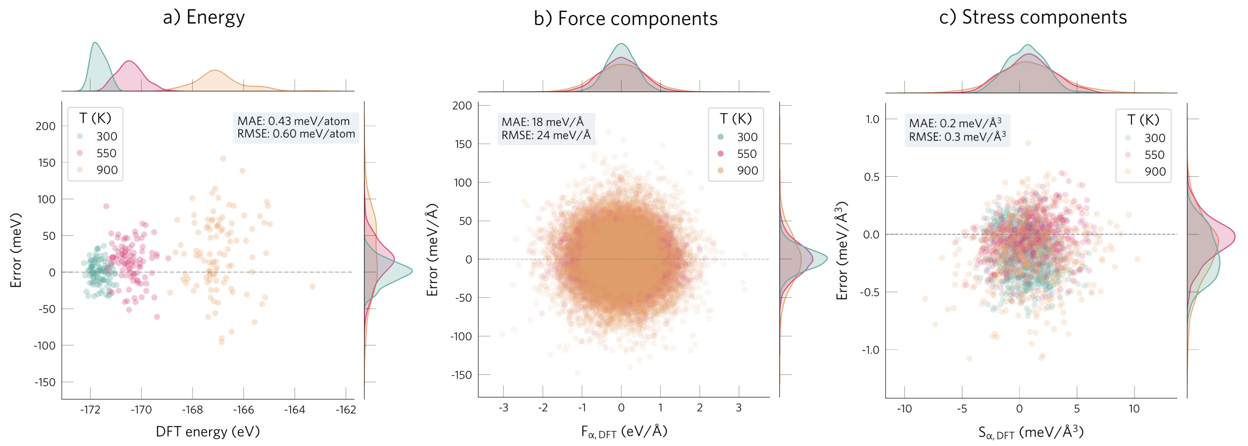

Supplementary Figure S3 Distribution of mean absolute and root mean squared errors (MAE, RMSE) for the test set of ${V_{\mathrm{Te}}}^{2+}$.

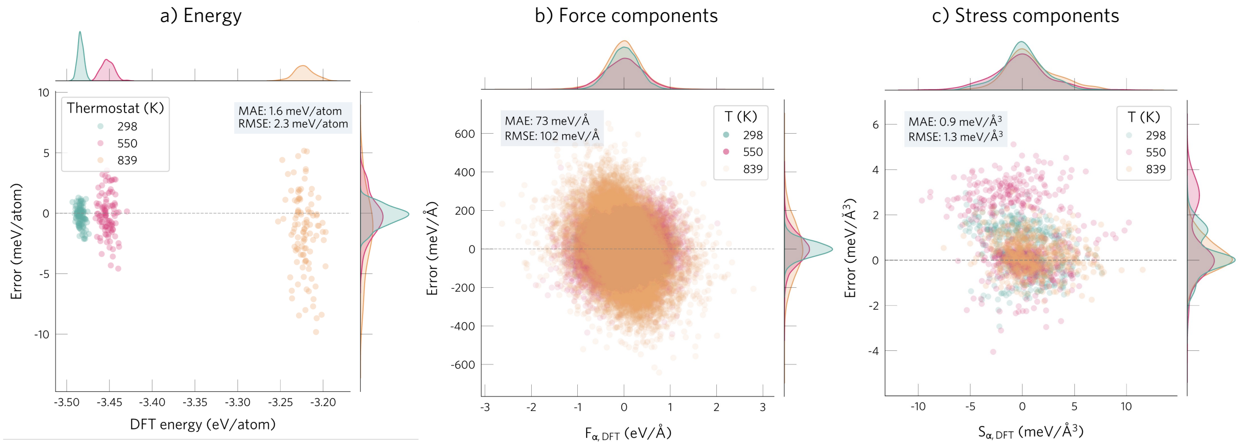

Supplementary Figure S4 Distribution of mean absolute and root mean squared errors (MAE, RMSE) for the test set of Te. Note that Te melts at 720 K, leading to larger errors for the liquid phase (T=900 K).

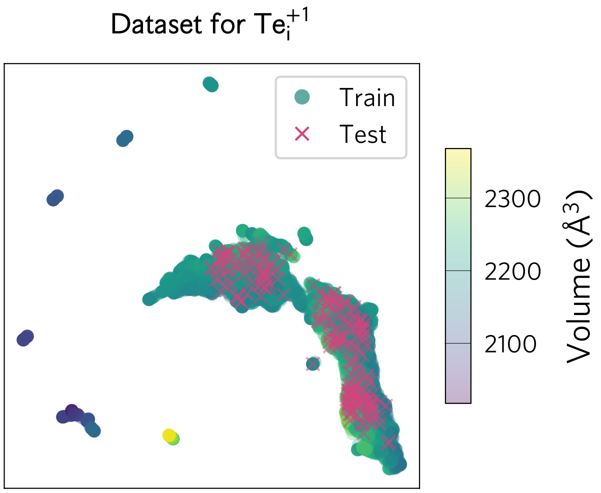

Supplementary Figure S5 Two-dimensional feature map for the configurations in the dataset of Te_i^{+1}. The configurations used for training are shown with circles, while the test ones are displayed with pink crosses. The isolated clusters of training datapoints correspond to compressed and expanded structures generated by scaling the equilibrium volume, which was necessary to ensure that the model could be applied with the quasiharmonic approximation. These regions were not included in the test set, which was designed to measure the accuracy of the model in typical application conditions (1 atm, 100-900 K), and accordingly expands over the configurations with equilibrium volumes at 1 atm. The good coverage of the test set over the training set feature space demonstrates its ability to quantify the accuracy of the model. Each configuration was encoded with its `DIRECT` descriptor (averaged over sites) and the dimensions were reduced using the Uniform Manifold Approximation, as implemented in the `UMAP` package[132].

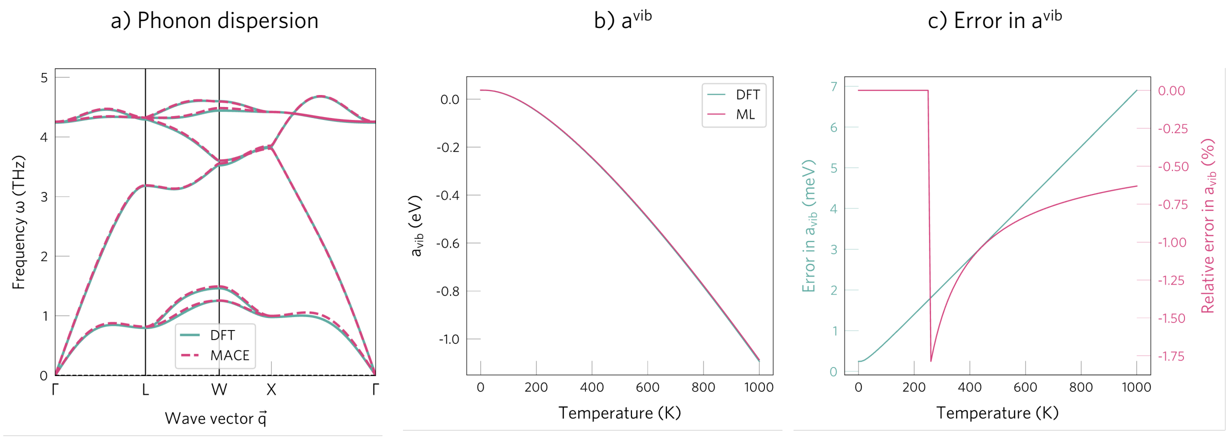

Supplementary Figure S6 Comparison of the harmonic phonon dispersion and vibrational free energy calculated with DFT and the `DIRECT` MLFF for bulk CdTe (primitive cell, 2 atoms). Note that the abrupt change in the relative error is caused by a^{vib} changing sign (i.e., division by 0 in $\Delta a = (a^{ML} - a^{DFT})/a^{DFT}$).

B. Learning curve

We analysed the learning curve for a model describing the behaviour of Te_i^{+1} at temperatures 100-900 K. We generated training sets with increasing number of configurations using the `DIRECT` method to sample the most diverse structures from the full dataset. This resulted in eight sets containing 50, 795, 1541, 2287, 3033, 3778, 4524 and 5270 configurations, which were used to train eight separate `DIRECT` models. By evaluating the performance of these models on the same test set (300 configurations, shown in Supplementary Figure S5), we can see that good accuracies ($MAE_E \leq 1$ meV/atom) can be achieved with only 50 configurations (Supplementary Figure S8), as long as these are sampled to maximise their diversity. However, this accuracy level is not enough to properly capture the small energy differences between the stable configurations of Te_i^{+1}, which only differ by $\Delta E(C_{2v} - C_s)_{DFT} = 18$ meV/supercell $= 0.3$ meV/atom. As demonstrated in Supplementary Figure S9, training sets with at least 1500 configurations are needed to accurately describe the barrier.

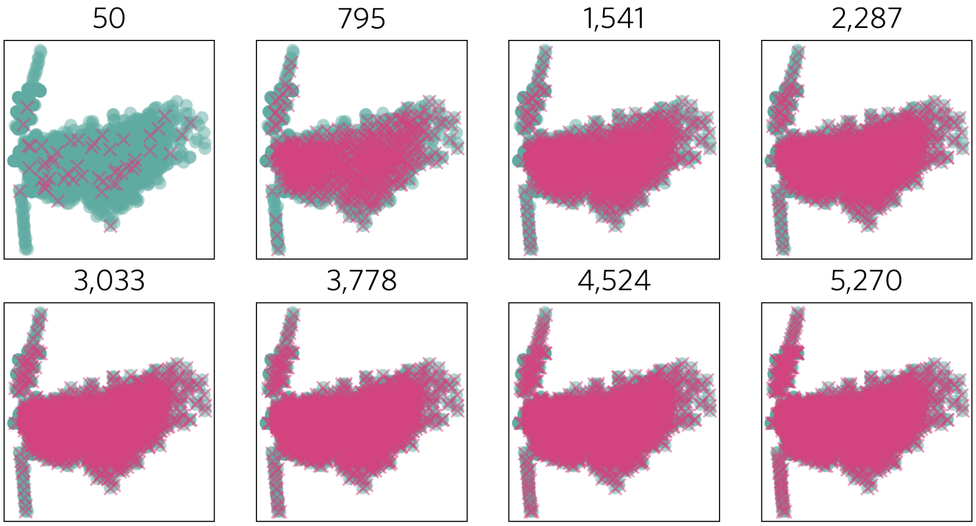

Supplementary Figure S7 Training sets of increasing size generated by sampling increasing number of configurations from the full dataset of 6016 Te_i^{+1} structures. Green circles illustrate the full dataset of configurations, while the selected structures are shown in pink crosses. Sampling was performed with the `DIRECT` algorithm[133]. Each structure was encoded with its `MACE` descriptor (averaged over sites) and the dimensions were reduced using Principal Component Analsysis, as implemented in the `maml` package[141].

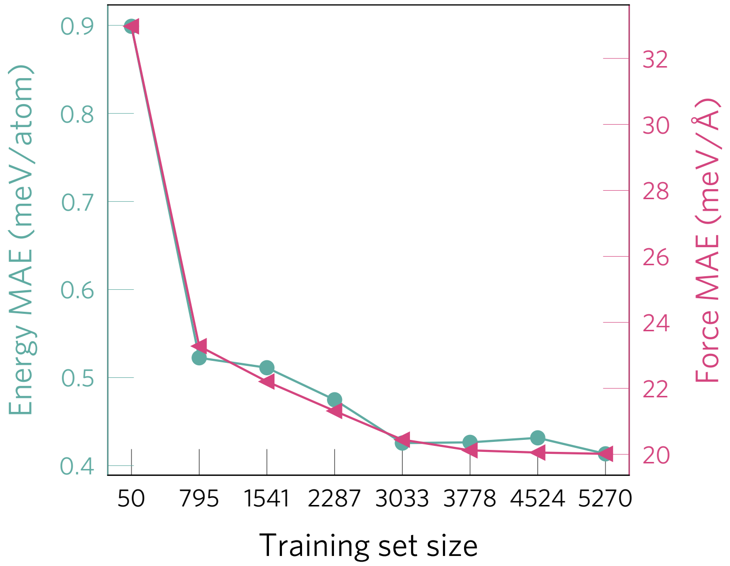

Supplementary Figure S8 Learning curve for the Te_i^{+1} machine learning force field, showing the energy and force mean absolute errors (MAE) on the test set for models with an increasing number of training configurations. The root mean square error shows a similar trend to the MAE. Models of acceptable accuracy ($MAE_E \leq 1$ meV/atom) can already be achieved with a small training set (50-1000 configurations), which is notable considering that the test set encompasses configurations from temperatures 300-900 K. The different training sets were generated by sampling the full dataset using the `DIRECT` algorithm[133] to ensure optimal coverage of the configurational landscape, as illustrated in Supplementary Figure S7.

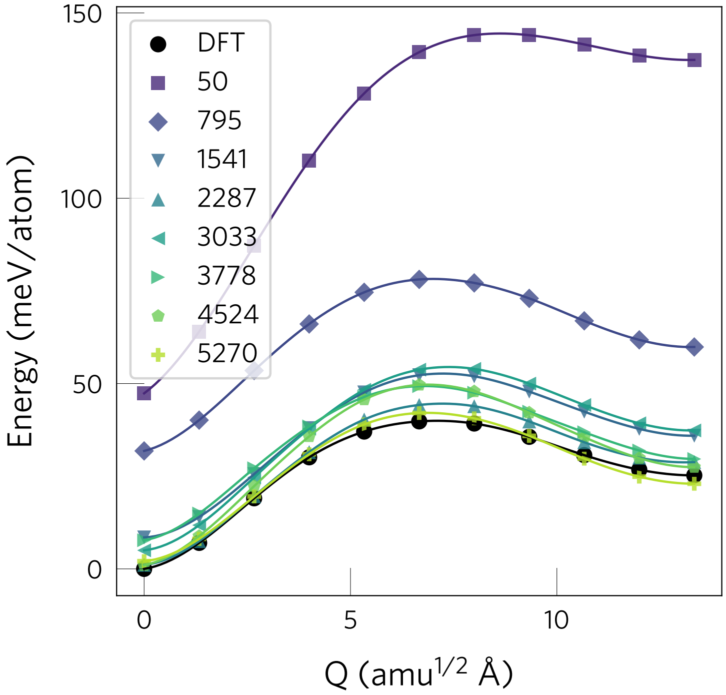

Supplementary Figure S9 Performance of the models with increasing training sizes on the energy barrier between the stable structures. Even for models with a low test error (MAE < 0.5 meV/atom), the errors in the barrier are significant. This is caused by i) the small energy differences between the configurations ($E_{barrier,DFT} \approx 40$ meV/supercell $= 0.6$ meV/atom, $\Delta E(C_{2v} - C_s)_{DFT} = 18$ meV/supercell $= 0.3$ meV/atom) and ii) the fact that the configurations in the training set are sampled to maximise diversity. This method samples most structures from the high energy region of the PES (and thus less structures from the 0 K path between the defect configurations). The legend denotes the number of configurations in the training set of each model, with the colormap ranging from dark purple to light green for increasing number of configurations. All energies are referenced to the DFT energy of the ground state configuration ($Q = 0$ amu$^{1/2}$Å).

C. Training machine learning force fields for defects

In this study, we trained separate MACE force fields for the pristine and defective supercells since this lead to higher accuracies. However, we have observed that this approach limits the defect models when applied to *larger supercells* than the ones used for training. While the models correctly describe the relative energies of the different configurations, they result in a systematic energy error. This is caused by how the MLFFs decompose the total energy into atomic contributions. When a model is trained only on *defect* configurations, part of the energy associated with the formation of the defect (e.g. one additional/missing atom and broken/reformed bonds) is spread over the energies of all atoms in the supercell. If the model is then applied to a larger supercell, the predicted atomic energies for bulk-like atoms do not correspond to the energy of an atom in a bulk-like environment, leading to a systematic energy shift (Supplementary Figure S10.b).

To solve this issue, one should train on *both* defect and pristine supercells. To illustrate this point, we trained three MACE models on three different datasets: i) 200 defect configurations of Te_i, ii) 100 defect (Te_i) and 100 pristine configurations and iii) 200 defect (Te_i) and 200 pristine configurations. Each dataset was sampled from the original training set ($2 \times 2 \times 2$ supercells) using the `DIRECT` method to maximise structural diversity. As illustrated in Supplementary Figure S10, while the model trained only on defect configurations leads to a smaller MAE when validating on configurations with the same number of atoms as the training structures ($2 \times 2 \times 2$ supercell, 65 atoms), it results in a systematic error when applied to larger supercells ($3 \times 3 \times 3$ (217 atoms) and $4 \times 4 \times 4$ (513 atoms)). This leads to a general conclusion when training MLFFs for point or extended defects: the training dataset should include both defect and pristine configurations if the models will be applied to larger defect supercells.

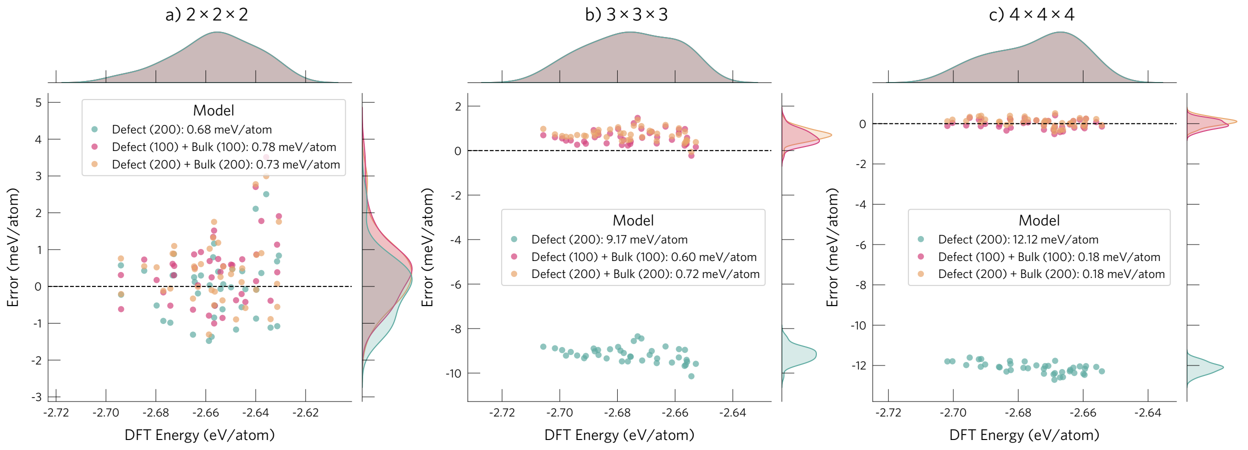

Supplementary Figure S10 Comparison of test errors between the energies predicted by the $\mathrm{Te_i}^0$ models and DFT, when validated on supercells of different sizes: a) $2 \times 2 \times 2$, b) $3 \times 3 \times 3$ and c) $4 \times 4 \times 4$. Different colours correspond to the MACE models trained on different datasets: i) only defective structures of $\mathrm{Te_i}^0$ (200 configurations), ii) 100 $\mathrm{Te_i}^0$ structures and 100 pristine structures and iii) 200 $\mathrm{Te_i}^0$ structures and 200 pristine structures. For each supercell size, the 40 test structures used for validation were sampled from heating runs (from 300 to 600 K with a heating rate of 0.7 K/ps) using the `DIRECT` method.

2. DYNAMIC ANALYSIS

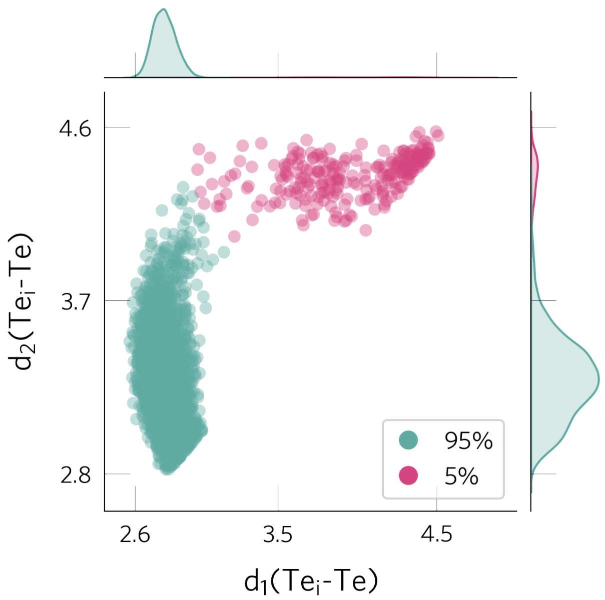

Supplementary Figure S11 Changes in the configuration of Te_i^{+1} at 300 K, quantified by monitoring the shortest Te_i-Te distances. While there is little variation in the shortest Te-Te bond (localised peak for d_1), the second shortest bond shows a wide variation ($d_2 = 2.8 - 4.0$ Å) — illustrating the change between the low-energy metastable configurations. Finally, there are some configurations where the interstitial occupies a significantly less favourable position without any Te-Te bonds (pink data points).

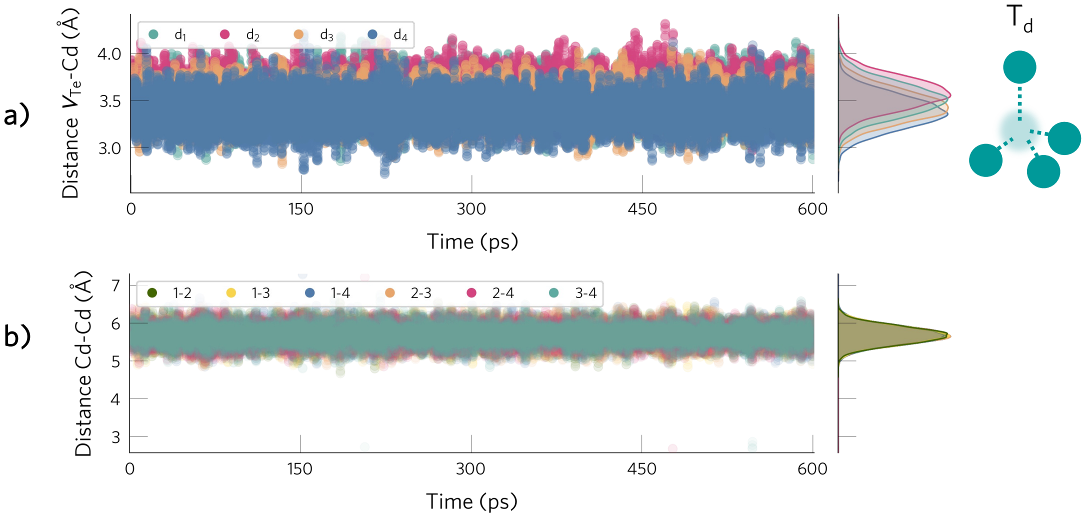

Supplementary Figure S12 Evolution of $V_{\mathrm{Te}}{}^{+2}$ at 1 atm and 300 K (NPT ensemble), which stays in its T_d configuration. a) Distance between the vacancy and its Cd neighbours, showing that all of them stay at a similar distance. b) Distance between the four Cd atoms neighbouring the vacancy, with these distances being similar and showing little variation with time.

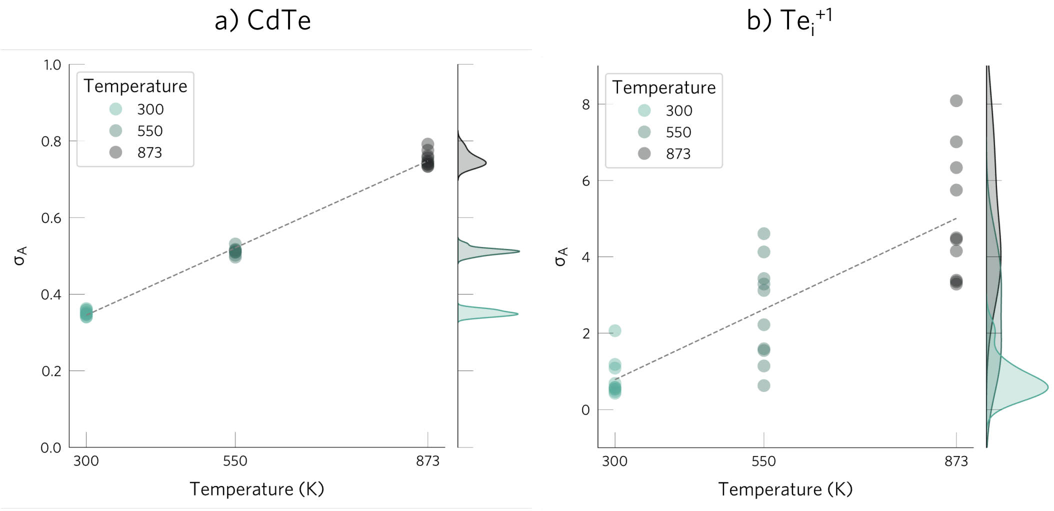

Supplementary Figure S13 Anharmonicity scores[87] for bulk CdTe and Te_i^{+1}, calculated on ten independent NPT trajectories (1 atm, T=300, 550, 873 K). The high anharmonicity scores of Te_i^{+1} are caused by its dynamic character (changes in configuration and position). For both bulk and the defect, the anharmonic character increases with temperature as the vibrational amplitude increases.

3. FREE ENERGIES

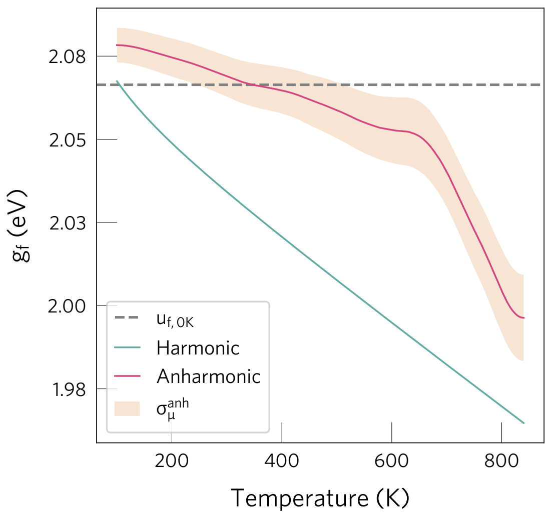

Supplementary Figure S14 Comparison of approximations for calculating the defect formation free energy, $g_f(T)$, of ${V_{\mathrm{Te}}}^{+2}$. For comparison, the formation internal energy, $u_f(0\ \mathrm{K})$, typically used in defect studies, is shown with a dashed grey line. Here we use $u_f(0\ \mathrm{K})$ instead of $u_f(0\ \mathrm{K}) - T(s_f^{spin} + s_f^{orient})$ since for ${V_{\mathrm{Te}}}^{+2}$ the terms s_f^{spin} and s_f^{orient} are zero (${V_{\mathrm{Te}}}^{+2}$ has no unpaired electrons and it keeps the original Tetrahedral site symmetry of the host crystal). This comparison shows that for ${V_{\mathrm{Te}}}^{+2}$, entropic effects are small and barely affect $u_f(0\ \mathrm{K})$ — with the thermal correction lowering $u_f(0\ \mathrm{K})$ by 0.08 eV, which accounts to 3.8% of $u_f(0\ \mathrm{K})$). This small effect agrees with its static behaviour at room temperature (Supplementary Figure S12). Note that the change in slope of g_f^{anh} is caused by the change in the free energy of Te, which melts at 704 K.

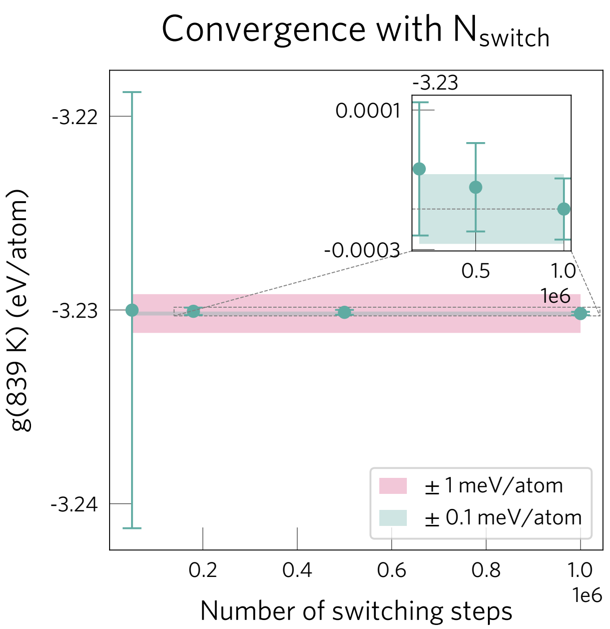

Supplementary Figure S15 Convergence of the temperature-dependent free energy of Te_i with respect to the number of switching steps used in the non-equilibrium thermodynamic integration simulations. The convergence is evaluated by comparing the values of the free energy at the end temperature (839 K). Note that differences below 0.1 meV/atom are achieved for $N_{\text{switch}} \geq 1.8 \times 10^5$ steps. The timestep was set to 2 fs and the uncertainties are determined by the mean standard error between 10 independent simulations performed for each value of N_{switch}.

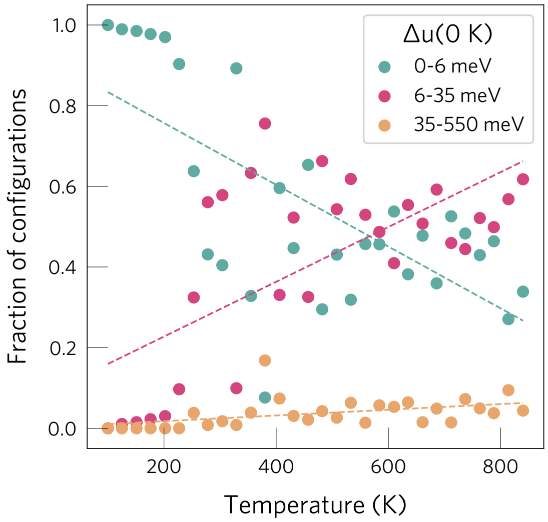

Supplementary Figure S16 Distribution of Te_i^{+1} in the ground state and metastable structures at different temperatures. The populations of the configurations are determined using the 'Inherent structures' formalism, by performing NPT MD simulations at different temperatures (80 ps, 1 atm), sampling 1600 equally-spaced configurations and relaxing them to their 0 K local minima using a conjugate gradient optimiser[25]. While at low temperatures (T > 200 K), the defect resides only in its lowest energy structure, above this temperature the population of the metastable configuration (i.e. the structure with higher internal energy) increases until it reaches the value of the ground state structure.

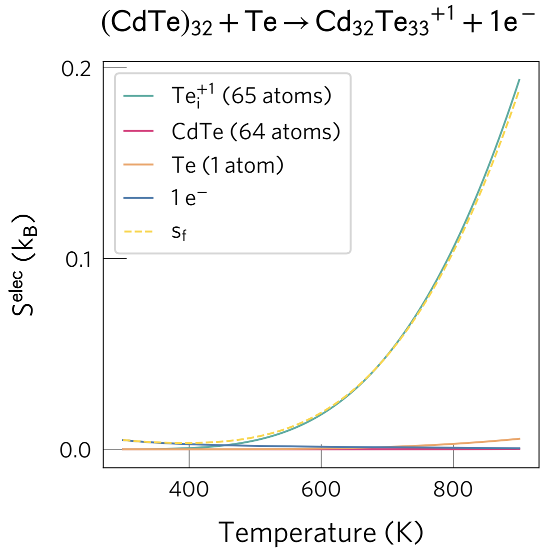

Supplementary Figure S17 Electronic entropy for the formation of Te_i^+. The increase in entropy is caused by the defect introducing an empty electronic level 0.7 eV above the valence band maximum.

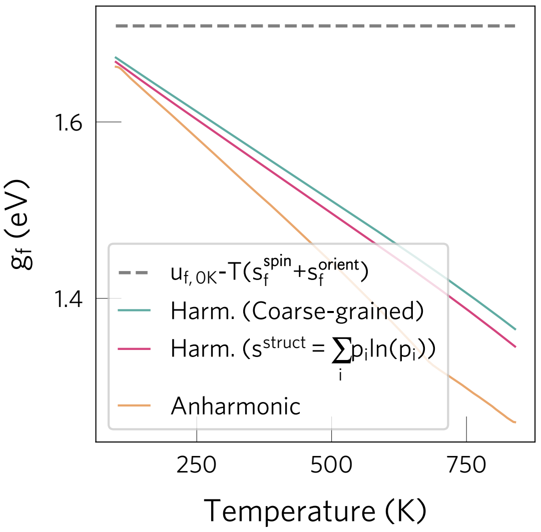

Supplementary Figure S18 Comparison of methods to calculate the structural or configurational entropy for the formation of Te_i^+. The coarse-grained approach uses Equation (16) from the main text while the second method uses Equation (11), as explained in the Methods. Both approaches result in a similar formation free energy.

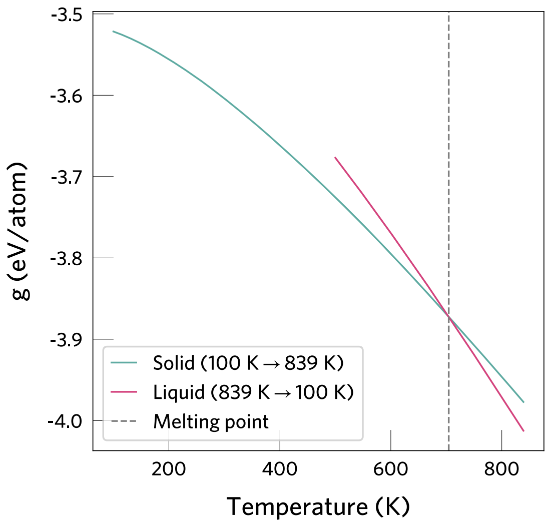

Supplementary Figure S19 Temperature variation of the Te free energy calculated with thermodynamic integration, showing the two independent integration paths and the resulting melting point (704 K), in reasonable agreement with the previously reported value of 722 K[123].
